# Large low-field magnetic enhancement in photoredox hydrodechlorination of *N*-heteroarene chlorides restricted by poor cage escape

**DOI:** 10.1093/nsr/nwag450

**Published:** 2026-07-24

**Authors:** Mingli Sun, Chenli Chen, Lingfang Chen, Shen Zhou, Jianyu Liu, Binghui Xue, Panchao Yin, Jiayi Liang, Shuming Bai, Jialong Jie, Hongmei Su, Song Gao, Linan Zhou

**Affiliations:** Spin-X Institute, School of Chemistry and Chemical Engineering, Guangdong-Hong Kong-Macao Joint Laboratory of Optoelectronic and Magnetic Functional Materials, State Key Laboratory of Luminescent Materials and Devices, South China University of Technology, Guangzhou 511442, China; College of Chemistry, Beijing Normal University, Beijing 100875, China; Beijing National Laboratory for Molecular Sciences, State Key Laboratory for Structural Chemistry of Unstable and Stable Species, Institute of Chemistry, Chinese Academy of Sciences, Beijing 100190, China; College of Science, National University of Defense Technology, Changsha 410003, China; College of Science, National University of Defense Technology, Changsha 410003, China; South China Advanced Institute for Soft Matter Science and Technology, Guangdong Basic Research Center of Excellence for Energy and Information Polymer Materials, State Key Laboratory of Luminescent Materials and Devices, South China University of Technology, Guangzhou 510641, China; South China Advanced Institute for Soft Matter Science and Technology, Guangdong Basic Research Center of Excellence for Energy and Information Polymer Materials, State Key Laboratory of Luminescent Materials and Devices, South China University of Technology, Guangzhou 510641, China; Beijing National Laboratory for Molecular Sciences, State Key Laboratory for Structural Chemistry of Unstable and Stable Species, Institute of Chemistry, Chinese Academy of Sciences, Beijing 100190, China; Beijing National Laboratory for Molecular Sciences, State Key Laboratory for Structural Chemistry of Unstable and Stable Species, Institute of Chemistry, Chinese Academy of Sciences, Beijing 100190, China; College of Chemistry, Beijing Normal University, Beijing 100875, China; College of Chemistry, Beijing Normal University, Beijing 100875, China; Spin-X Institute, School of Chemistry and Chemical Engineering, Guangdong-Hong Kong-Macao Joint Laboratory of Optoelectronic and Magnetic Functional Materials, State Key Laboratory of Luminescent Materials and Devices, South China University of Technology, Guangzhou 511442, China; Key Laboratory of Bioinorganic and Synthetic Chemistry of Ministry of Education, School of Chemistry, LIFM, IGCME, GBRCE for Functional Molecular Engineering, Sun Yat-sen University, Guangzhou 510275, China; Beijing National Laboratory of Molecular Science, Beijing Key Laboratory of Magnetoelectric Materials and Devices, College of Chemistry and Molecular Engineering, Peking University, Beijing 100871, China; Spin-X Institute, School of Chemistry and Chemical Engineering, Guangdong-Hong Kong-Macao Joint Laboratory of Optoelectronic and Magnetic Functional Materials, State Key Laboratory of Luminescent Materials and Devices, South China University of Technology, Guangzhou 511442, China

**Keywords:** photoredox, radical ion pairs, magnetic field effect, hyperfine coupling mechanism, cage escape

## Abstract

The efficiency of photoredox catalysis has long been hampered by rapid back electron transfer (BET) within the radical ion pairs (RIPs). While external magnetic fields can modulate the spin dynamics of RIPs to suppress BET, achieving a large magnetic field effect (MFE) at low field strengths has remained a formidable challenge. Herein, we report a remarkably large MFE, with a quantum yield enhancement exceeding 130% and a yield enhancement up to 160%, in the photoredox hydrodechlorination of *N*-heteroaryl chlorides under a weak magnetic field. This enhancement arises from the effective magnetic field modulation of the hyperfine-coupling-driven spin dynamics within triplet-born RIPs, particularly for systems with poor cage escape. By reversely applying a kinetic model developed in our previous work, we successfully deconvolve complex reaction pathways and quantitatively reproduce the intrinsic magnetic sensitivity, reconciling experimental observations with theoretical predictions in terms of half-saturation field. This work provides a new paradigm for photoredox transformation through spin modulation and demonstrates practical synthetic utility at high conversions, offering a powerful strategy for enhancing catalytic efficiency through accessible magnetic fields.

## Teaser

This study demonstrates over 100% magnetic field enhancement in both quantum and product yields for complex photoredox organic reactions, requiring only an easily attainable magnetic field achievable via permanent magnets.

## INTRODUCTION

Photoredox catalysis has emerged as a cornerstone of modern chemical synthesis, prized for its mild reaction conditions, high atom efficiency and environmental compatibility [1]. Its applications span a vast range of fields, including organic synthesis, energy conversion and biotherapy [2]. The canonical mechanism involves a single electron transfer (SET) from an excited-state photocatalyst to a substrate, generating a radical ion pair (RIP) that initiates the desired chemical transformation [3]. A persistent challenge, however, is the tendency of the geminate RIP to undergo rapid back electron transfer (BET) within the solvent cage, a process that dissipates energy and suppresses the product yield (Fig. 1a) [4]. Despite its fundamental importance, the efficiency of cage escape is frequently overlooked in the optimization of photocatalytic systems [4]. Prevailing strategies to mitigate deleterious BET have predominantly relied on harnessing triplet-state reactivity [5,6]. This approach exploits the fact that recombination from a triplet radical ion pair (^3^RIP) to the singlet ground state is a spin-forbidden process [3]. Capitalizing on this principle, researchers have widely employed strategies to promote efficient intersystem crossing (ISC), such as utilizing heavy-metal photocatalysts (e.g. Ru or Ir complexes) [7,8] or engineering organic photosensitizers with incorporated carbonyl groups [9] or heavy atoms [10]. Nevertheless, even for systems proceeding through the triplet pathway, the ^3^RIP can still undergo ISC to the ^1^RIP, creating a leakage pathway that enables considerable BET and thus diminishes the overall efficiency [11]. With advances in spin chemistry, external magnetic fields have emerged as a non-invasive, precisely tunable tool to modulate the spin dynamics of RIPs and, by extension, catalytic outcomes [12]. This approach presents a compelling alternative to traditional molecular engineering, especially for systems suffering from inherent poor cage escape

**Figure 1.**
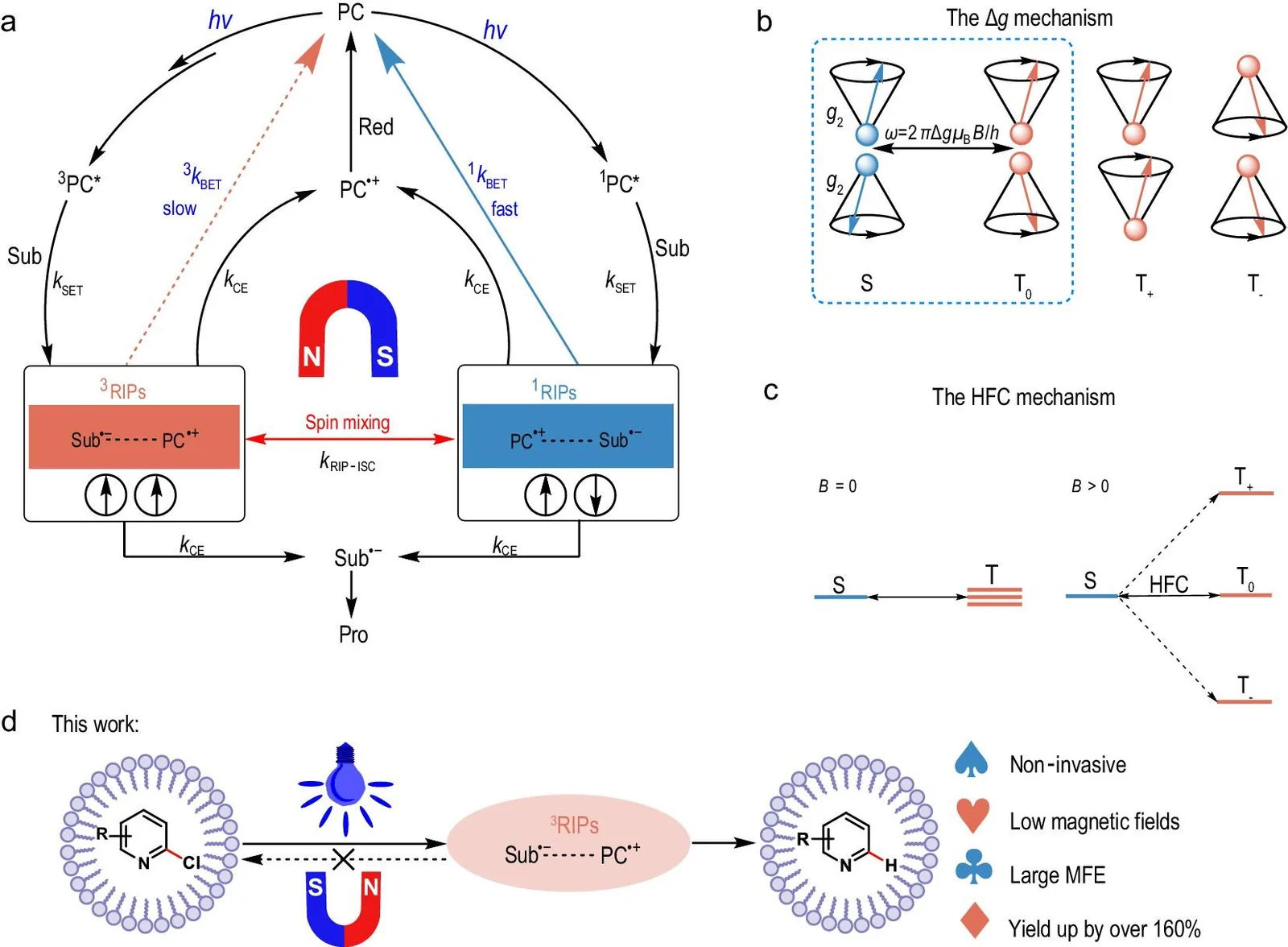
Magnetic field modulation of RIP dynamics in photoredox catalysis. (a) Competition between productive cage escape and unproductive BET for RIPs with different spin states during photoredox catalysis. (b) The Δ*g* mechanism. (c) The HFCM. (d) Photoredox hydrodechlorination of *N*-heteroaryl chlorides within micellar nanoreactors under a weak external magnetic field. HFC, hyperfine coupling; CE, cage escape.

[13]. Understanding the operative mechanism of such magnetic field effects (MFEs) is paramount for achieving a high MFE under low field strength, the latter of which is especially critical for practical catalytic applications. To date, two primary mechanisms have been identified for the magnetic field modulation of spin states in RIPs: the Δ*g* mechanism and the hyperfine coupling mechanism (HFCM) [14]. The Δ*g* mechanism, which promotes singlet–triplet (S↔T_0_) interconversion based on differences in radical *g*-factors (Fig. 1b), typically requires strong magnetic fields (>1 T) to elicit substantial effects in organic systems composed of light atoms [15]. This reliance on powerful, energy-intensive electromagnets renders the Δ*g* mechanism impractical for routine synthetic applications. Nevertheless, it should be noted that in specific organic systems exhibiting a large Δ*g*, the MFE behavior at low fields can be jointly governed by both the Δ*g* mechanism and the HFCM [16,17]. In contrast, the HFCM, which mediates interconversion between the singlet (S) and all three triplet sublevels (T_0_, T_+_ and T_−_), operates effectively under weak, practical magnetic fields (<0.1 T) [16,18]. In this regime, an applied field comparable to the hyperfine coupling strength is enough to lift the degeneracy of the triplet sublevels via Zeeman splitting, energetically isolating the T_±_ states from the singlet state and thereby suppressing hyperfine-induced ISC (Fig. 1c) [19]. Pioneering works by Turro, Hayashi and others established that micellar confinement can extend the lifetime of radical pairs, allowing sufficient spin evolution for MFEs to become observable [13,20–22]. However, these early investigations primarily focused on elucidating physical mechanisms using model probe molecules (e.g. ketones and quinones) [22,23], often with limited emphasis on synthetic utility or high-conversion transformations. Our recent study demonstrated that triplet charge recombination (TCR) in micellar nanoreactors can open a magnetic-field-sensitive pathway, achieving a positive MFE of up to ∼36% for photoredox synthetic purposes via HFCM [24]. While this work established the conceptual framework, the practical realization of large, synthetically transformative MFEs remains a formidable challenge. The modest magnitude observed previously is consistent with MFEs historically reported for diffusion-controlled systems (typically 0%–50%) [13,25], which has raised valid concerns about the general utility of magnetic modulation. This performance is hindered by kinetic constraints, wherein a complex interplay between competing pathways, including spin evolution, cage escape and BET, makes it difficult to guarantee a large MFE on the final product yield [3,26]. Consequently, this fundamental limitation has been a significant barrier, raising valid concerns about the general utility of magnetic modulation and preventing it from gaining traction as a practical tool within the synthetic community.

Herein, we report the realization of a large MFE, mediated by the HFCM, in the photocatalytic dechlorination of chloropyridines within a micellar system (Fig. 1d). Transformations of (hetero)aryl halides are foundational in medicinal chemistry and materials science, serving as indispensable tools for accessing privileged molecular scaffolds and enabling versatile late-stage diversification [27]. Among these, chlorinated heteroarenes offer compelling advantages in cost-effectiveness, commercial availability and bench stability compared with their brominated or iodinated counterparts. Despite the significant development of organic photosensitizers with exceptional reducing power like phenothiazine [28], the reductive activation of a C(*sp*2)–Cl bond on a heteroarene remains kinetically sluggish and intrinsically challenging [29]. Using the light-induced hydrodechlorination of chloropyridines as a model reaction, we demonstrate that a weak external magnetic field can remarkably enhance the quantum yield of heteroaryl C–Cl bond cleavage by over 130% and increase the yield of the hydrogenated product by 160%. This dramatic enhancement transcends the MFE reported in our earlier studies [24], signifies a fundamental shift in the reaction’s kinetic landscape, where the bottleneck moves from triplet-state population to the severe restriction of cage escape in the micellar environment, thus breaking the constraint of kinetic competition and achieving a large MFE. The specific chemical nature of micelles, along with micelle size, was shown to exert a pronounced influence on the MFE. Extending the kinetic model introduced in our prior work [24], we successfully utilized this framework to inversely deduce the spectroscopically unmeasurable cage-escape rate. Furthermore, it accurately captures the intrinsic magnetic field dependence, yielding a reasonable half-saturation field (*B*_1/2_) value in better agreement with theoretical predictions. Notably, the magnetic field enhancement remains pronounced even under high-conversion conditions, highlighting the robustness and synthetic utility of our spin-modulation strategy. This work not only presents an effective approach for harnessing exceptionally large MFEs in organic transformation but also pioneers a new paradigm for engineering advanced photoredox systems through the rational manipulation of spin dynamics in confined media.

## RESULTS AND DISCUSSION

### Optimization of reaction conditions for magnetic field enhancement

Traditional studies have shown that the magnitude of MFEs is governed by a complex interplay among competing microkinetic steps of key RIP intermediates, including molecular diffusion, electron transfer and spin evolution, within the reaction mechanism [30]. These microkinetic processes are themselves dictated by key system parameters, such as the reaction medium [31], the electronic structures of the photocatalyst and substrate [32] and hyperfine interactions [33,34]. We first investigated the MFEs of the model reaction in different solvent systems (Fig. 2a). Among the conventional organic solvents (Fig. 2b), only the highly polar dimethyl sulfoxide (DMSO) (∼2.1 cP) afforded a detectable reaction, with a rate of 0.45 mM min^−1^. However, this rate remained essentially unchanged upon the application of a 3000 G magnetic field. The absence of an MFE in regular solvents is common, as the short lifetime of RIPs in low-viscosity environments hinders effective magnetic modulation of spin-state evolution [34]. This was verified using the high-viscosity solvent, polyethylene glycol 400 (PEG-400) (∼30.3 cP), which exhibited a pronounced MFE of 19.3%, with the reaction rate increasing from 0.32 mM min^−1^ at zero field to 0.39 mM min^−1^ at 3000 G.

**Figure 2.**
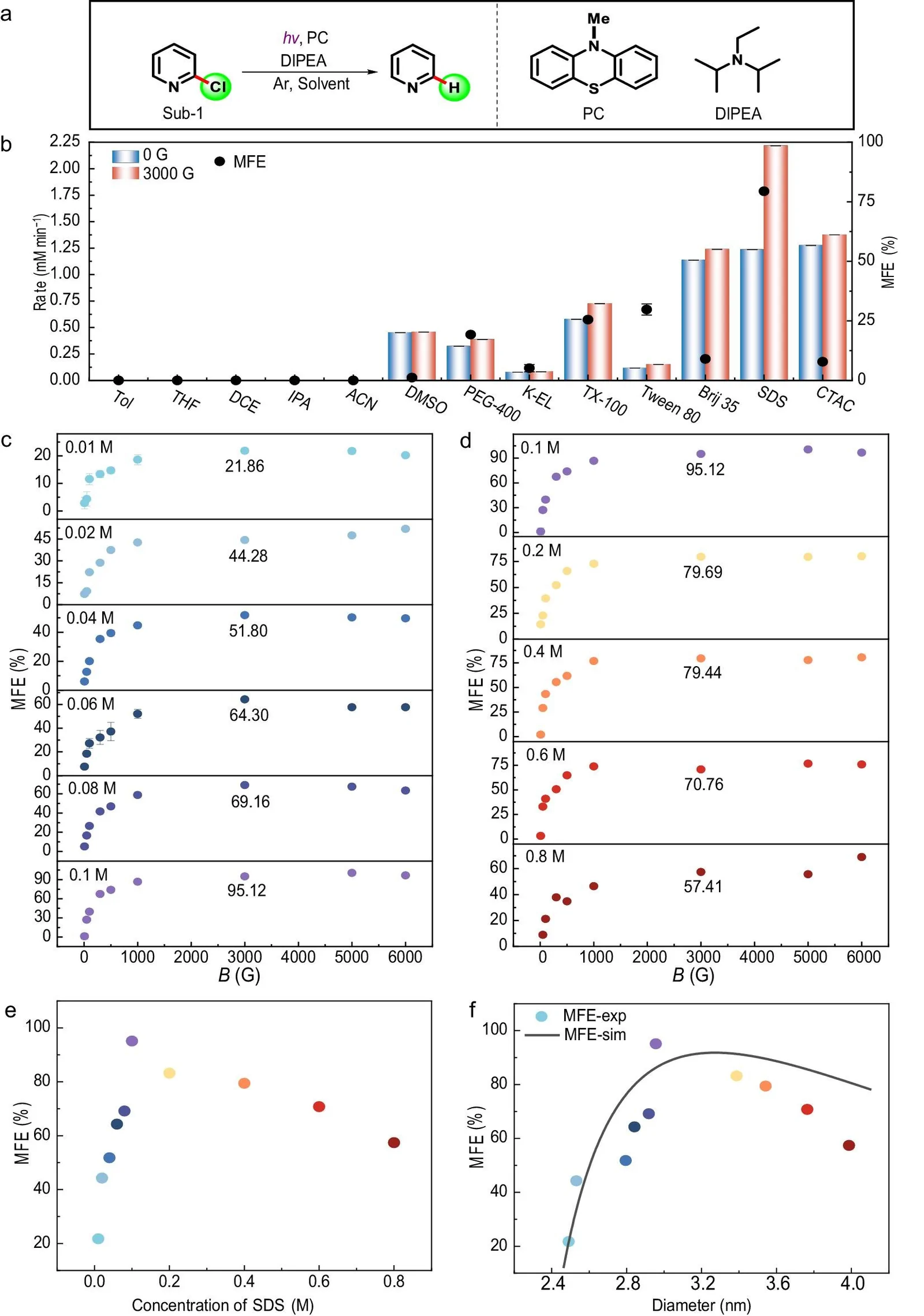
Optimization of reaction conditions for magnetic field enhancement. (a) The model reaction was conducted by irradiating with a continuous wave (CW) laser (375 nm, 145 mW) at room temperature in solvent (300 μL) under Argon, containing 50 mM 2-chloropyridine (Sub-1), 5 mol% 10-methyl-10*H*-phenothiazine (PC) and 2.0 equivalents of *N,N*-diisopropylethylamine (DIPEA). (b) Initial reaction rates and the corresponding MFE in various reaction media. (c) Dependence of the MFE on SDS concentration in the 0.01–0.1 M range. (d) Dependence of the MFE on SDS concentration in the 0.1–0.8 M range. (e) Correlation between the saturation MFE and the concentration of SDS. (f) Theoretical simulation of the dependence of the MFE on the micellar cage size. Reaction conditions are detailed in Table S7. MFE = [(Rate(B) − Rate(0))/Rate(0)] × 100%, where Rate(B) and Rate(0) represent the initial reaction rates in the presence and absence of an external magnetic field, respectively.

In addition to tuning solvent viscosity, micellar confinement provides another effective means to prolong the lifetime of RIPs. We therefore systematically investigated how distinct micellar systems—both nonionic and ionic—influence photocatalytic performance and respond to an applied magnetic field (Fig. 2b). To determine micellar dimensions, we specifically employed small-angle X-ray scattering (SAXS), as conventional dynamic light scattering is unreliable at the high surfactant concentrations employed in this study (refer to ST-1 of the Supplementary data for the details) [35]. First, the micelles exhibit different intrinsic activity under zero-field conditions, which is attributed to the unique environment that different micelles provide. An applied weak magnetic field is not expected to alter these physical and microenvironmental properties. Therefore, the observed relative enhancement is expected to originate from the magnetic modulation of spin dynamics. Among nonionic surfactants, K-EL (4.17 nm) and Brij 35 (2.86 nm) produced only modest MFEs of 5.2% and 9.0%, respectively, whereas TX-100 (2.87 nm) and Tween 80 (3.32 nm) demonstrated substantially larger effects of 25.5% and 29.8%. This disparity cannot be attributed to micelle size alone: TX-100 and Brij 35 form micelles of nearly identical diameter, yet the MFE in the TX-100 system is 3-fold greater. Ionic micelles accentuate this contrast. Anionic sodium dodecyl sulfate (SDS) micelles (3.54 nm) delivered a remarkably high MFE of 79.4%, while cationic cetyltrimethylammonium chloride (CTAC) system with similar micellar size (3.89 nm) displayed an effect of only 7.8%. These observations indicate that, beyond physical confinement, specific chemical interaction—dictated by the surfactant’s molecular structure—appears to play a dominant role in modulating the magnetic responsiveness. For instance, the opposite headgroup charge of the ionic micelles could exert completely distinct effects on the microkinetics of the RIP. Similarly, the aromatic moieties in TX-100 can engage in noncovalent *π*–*π* interactions with photocatalytic intermediates such as PC^•+^ and Sub^•−^, thereby influencing the diffusion escaping kinetics. To ensure the reliability of these prominent MFEs, particularly in the highly efficient SDS system, comprehensive control experiments were conducted. The results ruled out macroscopic viscosity and local photothermal heating as contributors, thereby confirming a purely spin-chemical origin (Table S1 and Figs S1 and S2). It is noteworthy that the observed field-dependent behavior, characterized by a positive enhancement of reaction yield that saturates at low fields (Fig. S3), is most consistent with the HFCM of triplet-born RIPs, which will be further verified below.

Although surfactant chemistry is a primary determinant of the MFE, micelle size remains a critical, tunable parameter that can reinforce or attenuate these effects. To further investigate the effect of micelle size and identify conditions that maximize the MFE, we selected SDS, the most MFE-efficient surfactant, for a detailed, size-focused study. SAXS measurements revealed an approximately linear dependence of SDS micelle size on concentration (refer to ST-1 of the Supplementary data for the details). In the 0.01–0.1 M concentration range, the micelles remain spherical, and their hydrodynamic diameter increases from 2.49 to 2.96 nm (Fig. S4 and Table S2). This size growth is directly correlated with enhanced MFEs (Fig. 2c). At concentrations above 0.2 M, however, the micelles transform into ellipsoids (Fig. S5 and Table S3), and the MFE decreases as micelle size continues to increase (Fig. 2d). As summarized in Fig. 2e, the magnitude of the saturated MFE exhibits a peak-like dependence on the concentration of SDS, with a maximum of 95% at 0.1 M (2.96 nm). These observations point to the existence of an optimal cavity size that balances spatial confinement with effective separation of RIPs. This optimum can be rationalized as a trade-off between ensuring spin coherence of RIPs and maintaining a weak exchange interaction (*J*). In excessively small micelles, extreme spatial confinement forces the radicals into close proximity. This results in a large *J* that greatly exceeds the Zeeman splitting energy, thus decoupling the spin evolution within the RIPs from the influence of the external magnetic field [30]. Conversely, in overly large micelles, the reduced re-encounter probability at large separations diminishes the efficiency of the magnetic field modulation. Consequently, the maximum MFE occurs at an intermediate size, where both the lifetime and spin interactions of RIPs are optimized. This enables the hyperfine interaction to efficiently drive ISC between degenerate singlet and triplet states, thus be effectively modulated by the external magnetic field via the HFCM.

To further substantiate this interpretation, we performed a theoretical simulation on the magnetic field responsiveness of our system (Fig. 2f). Our model introduces a cutoff distance, *r*_c_. For inter-radical distance, *r*, below *r*_c_, the spin evolution of the RIP is assumed to be magnetic field silent owing to the large spin exchange interaction. While for distance above *r*_c_, the magnetic field sensitivity is modeled to decay with distance as *r*^−6^, reflecting the distance dependence of spin correlation within the RIPs [36] (refer to ST-2 of the Supplementary data for the details). As expected, the optimal micellar cage size depends on the cutoff distance (Fig. S9) and the best agreement with experiment is obtained for *r*_c_ ≈ 1.73 nm. Compared to literature reports, this derived value is larger than the 0.6–0.8 nm range generally reported for free-diffusion systems in polar solvents [18] and is comparable to the 1.5–2.5 nm range observed for covalently linked biradicals [37,38]. Such an intermediate *r*_c_ is rationalized by two complementary factors. First, the micellar cage provides a form of soft confinement, which situates the system between the unrestricted diffusion of free radical pairs and the rigidly fixed geometry of covalent biradicals. Second, the non-polar environmental nature of the micellar core offers poor energetic stabilization for the charged RIP, resulting in a larger 2 *J* at any given distance compared to polar solvents [19]. Consequently, a greater inter-radical separation is required for the exchange interaction to fall within the magnetically responsive regime where hyperfine interactions dominate. The fact that our obtained *r*_c_ value reflects this physical reality provides compelling validation for our model and interpretation.

### Elucidation of reaction mechanism and understanding of magnetic field effect

To elucidate the reaction mechanism, femtosecond transient absorption (fs-TA) spectroscopy was conducted to study the light-induced electron transfer dynamics of PC in SDS micelles. Figure 3a shows the fs-TA spectra of PC following 355 nm photoexcitation. Immediately after the pump pulse, two broad excited-state absorption bands appeared at ∼390 and ∼650 nm. These bands, attributed to the singlet excited state of PC (^1^PC*), both reached their maxima within the instrumental response time (∼200 fs). These bands exhibit a slow decay on the nanosecond timescale, concurrent with the appearance of a new absorption band at ∼460 nm. This eventual spectral shape at 6 ns with the characteristic 460 nm band being indicative of a longer-lived transient species (Fig. 3b). This absorption profile closely matches the previously reported spectrum for the excited-state triplet of PC (^3^PC*) [39–42]. Through global fitting of the transient evolution, a time constant of 6.7 × 10^8^ s^−1^ (1.5 ns) was obtained, corresponding to the decay of ^1^PC*. This value represents the upper limit of ISC rate constant. Additionally, the evolution-associated difference spectra (EADS) derived from the global fit align well with the observed spectral shapes, reinforcing the validity of the fit (Fig. 3c and d).

**Figure 3.**
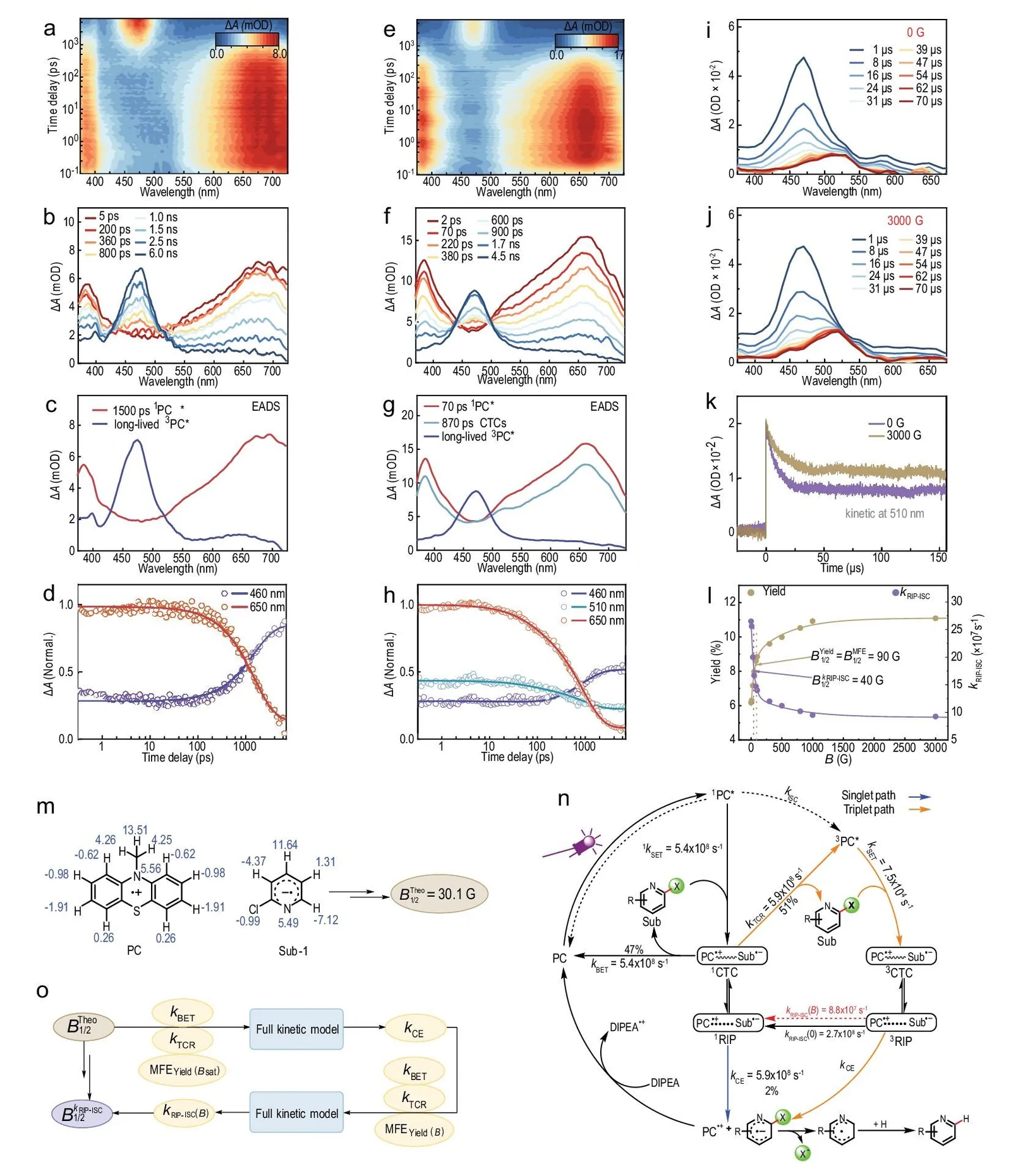
Mechanistic elucidation and kinetic analysis of the MFE. fs-TA spectra experiments using 355 nm laser excitation with reaction conditions of 5 mM 10-methyl-10*H*-phenothiazine (PC) and 50 mM DIPEA. (a, e) Two-dimensional (2D) contour map of femtosecond transient absorption in SDS (a) without or (e) with 50 mM 2-chloropyridine (Sub-1). (b, f) Transient absorption spectra (TAS) at typical delay times from panels (a) and (e), respectively. (c, g) EADS obtained from the global analysis of the transient spectra (a) and (e), respectively. (d, h) Time traces (dots) at typical wavelengths slicing from the 2D contour maps (a) and (e), respectively, together with the fits (lines) from the global analysis. ns-TA spectrum experiments using 355 nm laser excitation with reaction conditions of 5 mM PC and 50 mM Sub-1. (i, j) TAS at typical delay times under (i) 0 G and (j) 3000 G, respectively. (k) Comparison of the kinetic curves of PC^•+^ at 510 nm under 0 and 3000 G. (l) Comparison of the half-saturation field between the value derived from the overall reaction yield ($B_{{\mathrm{1/2}}}^{{\mathrm{Yield}}}$) and the theoretical prediction ($B_{{\mathrm{1/2}}}^{{\mathrm{Theo}}}$). (m) The theoretical half-saturation field ($B_{{\mathrm{1/2}}}^{{\mathrm{Theo}}}$) calculated from the hyperfine coupling constants of the PC^•+^ and the Sub-1^•−^. The detailed calculation formula is provided in the ST-4 of the Supplementary data. (n) The comprehensive kinetic model. Note that the spectroscopically determined kinetic constants are pseudo-first-order rate constants at a fixed substrate concentration of 0.05 M. (o) The half-saturation field ($B_{{\mathrm{1/2}}}^{{k}_{{\mathrm{RIP - ISC}}}}$) derived from the intrinsic spin interconversion rate extracted using the kinetic model.

In contrast, the fs-TA spectra of PC in the presence of Sub-1 differ significantly from those of PC alone (Fig. 3e–h). The interaction with Sub-1 notably accelerates the decay of ^1^PC*, accompanied by the emergence of a new spectral feature within 200 ps. It suggests that the SET event occurs between the ^1^PC* and the substrate within hundreds of picoseconds. For the new spectral feature: in addition to the pronounced absorption bands at 390 and 650 nm, a small shoulder appeared around 510 nm, which was assigned to the formation of the charge transfer complex (CTC) composed of the PC^•+^ and Sub-1^•−^. Interestingly, during the decay of the CTC, only one long-lived species, ^3^PC*, was detected, characterized by an absorption band near 460 nm. This suggests that, in addition to BET to the ground state, the CTC underwent a TCR to generate the localized triplet state (^3^PC*) [43]. Using the extinction coefficients of ^1^PC* and ^3^PC* estimated from the transient absorption data (refer to ST-3 and Fig. S10 of the Supplementary data for the details), the relative rates of these two decay pathways of the CTC were further quantified through global fitting (*k*_BET_ = 5.4 × 10^8^ s^−1^, *k*_TCR_ = 5.9 × 10^8^ s^−1^). Notably, no detectable signal corresponding to PC^•+^ at 510 nm was observed within the detection limit of the instrument, highlighting the unusually slow cage-escape kinetics of the CTC relative to its other decay pathways. This behavior stands in stark contrast to our previous investigation on chlorobenzonitriles, where rapid cage escape was readily observed [24]. Interestingly, the signal for the cage-escape product, PC^•+^, was undetectable not only in the confined micellar system but also across various conventional homogeneous organic solvents (Figs S11–S13). This indicates that the low cage-escape efficiency of the CTC is a general phenomenon rather than a characteristic specific to the micellar environment. These observations suggest that the sluggish diffusive separation of the CTC is a unique feature of chlorinated *N*-heteroarene substrates, potentially accounting for the low reaction efficiency commonly observed in conventional organic solvents.

As expected, micellar confinement enriches the substrate concentration around the photocatalyst [44], accelerating the chemical quenching of the ^3^PC* and promoting its entry into the catalytic cycle. This kinetic enhancement was confirmed by nanosecond transient absorption (ns-TA) spectroscopy, which revealed that bimolecular quenching rate constant in the micellar system (1.5 × 10^6^ M^−1^ s^−1^) is an order of magnitude greater than that in DMSO (1.5 × 10^5^ M^−1^ s^−1^) (Fig. S14). In particular, the formation of free photosensitizer radical cation PC^•+^ was successfully detected along this triplet reaction pathway (Fig. 3i). This observation is likely attributed to the longer lifetime of triplet charge-transfer complex (^3^CTC) compared to its singlet counterpart (^1^CTC), which, as revealed above by fs-TA, rapidly decays through BET and TCR processes. Having identified PC^•+^ as a key intermediate of the triplet pathway, we then investigated its behavior under a magnetic field. We explicitly observed a marked enhancement in the concentration of free PC^•+^ escaping from ^3^CTC under a 3000 G magnetic field (Fig. 3j and k). In contrast, no such effect was observed in DMSO (Fig. S15). Such magnetic-field-enhanced yield of escape radicals from triplet-born RIPs, in conjunction with the previously noted saturation of the MFE at low field strengths, provides compelling evidence that HFCM is the most plausible underlying mechanism for the observed MFE in this system. Specifically, under an external magnetic field, the ^3^RIP with weak exchange interactions undergoes Zeeman splitting into three sublevels (T_0_, T_+_ and T_−_) [19,45]. This splitting suppresses the triplet-to-singlet ISC rate of RIPs (*k*_RIP-ISC_) so that the probability that ^3^RIPs escape from the solvent cage prior to conversion to ^1^RIPs, which can be annihilated via charge recombination, is significantly increased [13,46].

The half-saturation field, *B*_1/2_, is a critical parameter in describing MFEs based on HFCM [33,47,48], representing the field strength at which the effect reaches half of its maximum and serving as a direct measure of the effective hyperfine interaction strength within the RIP. In our system, however, the *B*_1/2_ derived from the Yield*-B* response curve (Fig. 3l)—denoted as $B_{{\mathrm{1/2}}}^{{\mathrm{Yield}}}$ = 90 G—is anomalously larger than the theoretical value predicted by Weller’s equation [49] ($B_{{\mathrm{1/2}}}^{{\mathrm{Theo}}}$ = 30 G; Fig. 3m), which is more consistent with typical hyperfine interaction strengths in organic radicals. The origins of this significant deviation are multifaceted. First, we considered the limitations of Weller’s semi-empirical equation. As emphasized by Wong *et al.*, the commonly used Weller approximation neglects the anisotropic components of hyperfine interaction and typically underestimates the half-saturation field for restricted radical pairs. Nevertheless, such underestimation is usually modest (∼25%) and cannot by itself account for the much larger *B*_1/2_ we observe experimentally [47]. Second, we evaluated the potential role of spin relaxation. While spin relaxation mechanisms, such as dephasing driven by diffusive modulation of exchange interactions, can influence magnetic sensitivity in confined media, these processes typically occur on the microsecond timescale [50,51]; given the nanosecond-scale lifetime of the RIPs in our system, relaxation dynamics are unlikely to be the dominant factor in broadening the magnetic field response. We hypothesize that this discrepancy arises mainly because the measured yield is the composite outcome of multiple competing kinetic steps, rather than a direct reflection of *k*_RIP-ISC_, whose field-dependent behavior is the true measure of the system’s intrinsic magnetic sensitivity.

To obtain the half-saturation field of the RIPs’ ISC rate, $B_{{\mathrm{1/2}}}^{{k}_{{\mathrm{RIP - ISC}}}}$, we employed the comprehensive kinetic model developed in our previous work [24] (based on the mechanism proposed in Fig. 3n; refer to ST-4 of the Supplementary data for details), but in a reversed strategy: premised on the validity of the model, we deduced the unknown kinetic parameters. The model yields an explicit equation for the MFE on product yield, MFE_Yield_(*B*), as a function of four elementary kinetic constants: *k*_CE_ (cage-escape rate constant), *k*_BET_, *k*_TCR_ and the field-dependent *k*_RIP-ISC_(*B*). First, the equation shows that MFE_Yield_(*B*) is inversely correlated with the *k*_CE_. This is because a lower intrinsic cage-escape efficiency amplifies the relative impact of the magnetic field on suppressing the competing ISC pathway and funneling the RIPs that otherwise recombined toward the cage-escape channel (see decay pathways of ^3^RIP in Fig. 3n). Directly fitting this equation at arbitrary fields is mathematically underdetermined due to the presence of two unknown variables: *k*_CE_ and *k*_RIP-ISC_(*B*). To resolve this ambiguity without relying on multi-parameter free fitting, we utilized the saturated magnetic field condition (*B* ≥ *B*_sat_) to quantitatively determine *k*_CE_. First, the RIP ISC rate under the saturated magnetic field, *k*_RIP-ISC_(*B*_sat_), can be theoretically determined. Specifically, the theoretical half-saturation field ($B_{{\mathrm{1/2}}}^{{\mathrm{Theo}}}$), calculated from molecular hyperfine couplings via Weller’s equation [49], is used to determine the zero-field ISC rate constant [*k*_RIP-ISC_(0)] based on the well-established semiclassical equation of the radical pair mechanism [14,34]. Under high-field saturation, Zeeman splitting decouples the triplet sublevels, constraining *k*_RIP-ISC_(*B*_sat_) to one-third of its zero-field value. Subsequently, by substituting this theoretically estimated *k*_RIP-ISC_(*B*_sat_), the experimentally measured saturated MFE [MFE_Yield_(*B*_sat_)] and the two independently determined kinetic constants (*k*_BET_ and *k*_TCR_) into the kinetic equation, *k*_CE_ becomes the sole unknown and can be reliably quantified. With *k*_CE_ established, we can then derive the *k*_RIP-ISC_ value at fields other than the saturated field by repeatedly performing the same analysis using the known MFE_Yield_(*B*), *k*_CE_, *k*_BET_ and *k*_TCR_ (Fig. 3o). Following these procedures, we determined the cage-escape rate constant to be *k*_CE_ = 2.3 × 10^7^ s^−1^. This low value, corresponding to a mere 2% branching ratio of ^1^CTCs for escape pathway, is consistent with the non-detection of the escaped PC^•+^ in our ultrafast spectroscopy measurement. Crucially, the half-saturation field derived from *k*_RIP-ISC_(*B*), $B_{{\mathrm{1/2}}}^{{k}_{{\mathrm{RIP - ISC}}}}$ (40 G), shows better agreement with the theoretical prediction (30 G). This confirms that the elementary process of spin interconversion, not the composite reaction yield, serves as the true intrinsic descriptor of the MFE. The competition among multiple kinetic steps, especially the anomalously limited cage escaping kinetics, is likely responsible for the anomalously high half-saturation field observed in the experimental yield. Notably, the minor deviation between $B_{{\mathrm{1/2}}}^{{\mathrm{Theo}}}$ and $B_{{\mathrm{1/2}}}^{{k}_{{\mathrm{RIP - ISC}}}}$ can be reasonably attributed to the inherent limitations of the Weller’s equation [47].

### Substrate scope of magnetic field effects

To assess the generality of the MFE and elucidate the influence of substituents, we systematically examined the reactivity of various chloropyridine derivatives under both zero field and a 3000 G field, revealing clear substituent-dependent trends (Fig. 4a). Note that 0.4 M SDS was applied hereinafter to ensure complete solubilization of all substrates. The experimental results demonstrated that introduction of electron-donating groups (EDGs) tended to enhance the MFE compared to electron-withdrawing groups (EWGs) at the same position. For instance, methyl substitution at the C3 position (Sub-2) increased the MFE to 120% compared to the unsubstituted 2-chloropyridine (Sub-1, 81%), while introduction of –CF₃ (Sub-3) reduced it to 73%. A similar trend was observed at the C5 position, where 2-chloro-5-methylpyridine (Sub-5) and 2-chloro-5-trifluoromethylpyridine (Sub-6) exhibited MFEs of 131% and 90%, respectively. The same correlation held for C6 substitution: 2-chloro-6-methylpyridine (Sub-8) displayed an MFE of 98%, whereas 2-chloro-6-fluoropyridine (Sub-9) dropped to 56%. Interestingly, methyl substitution at C4 exerted only a marginal influence, with 2-chloro-4-methylpyridine (Sub-4) showing an MFE of 78%, comparable to the unsubstituted substrate, implying that the MFE may be relatively insensitive to electronic effects at the C4 position. Similar behavior was also observed for the 4-chloropyridine scaffold: EDG substitution at the C2 position (Sub-10) raised the MFE to 103%, while EWG substitution (Sub-11 and Sub-12) reduced it drastically to 16% and 34%, respectively. Notably, the introduction of a weak electron-donating -CH₂OH group at C5 (Sub-7) resulted in a marked decrease in the MFE to 41%, a phenomenon that might be associated with the higher polarity of hydroxyl group. Presumably, the introduction of EDGs increases the negative charge density on the pyridine ring radical anion. This, in turn, can strengthen the electrostatic attraction within the RIP and likely lead to a more tightly bound configuration that ultimately suppresses cage escape. Regardless of the functional groups, these *N*-heterocyclic substrates generally exhibit higher MFEs compared to the aromatic ring systems reported in our previous work with similar hyperfine interaction strengths [24], highlighting the unique low cage-escape characteristic of the *N*-heteroarene system.

**Figure 4.**
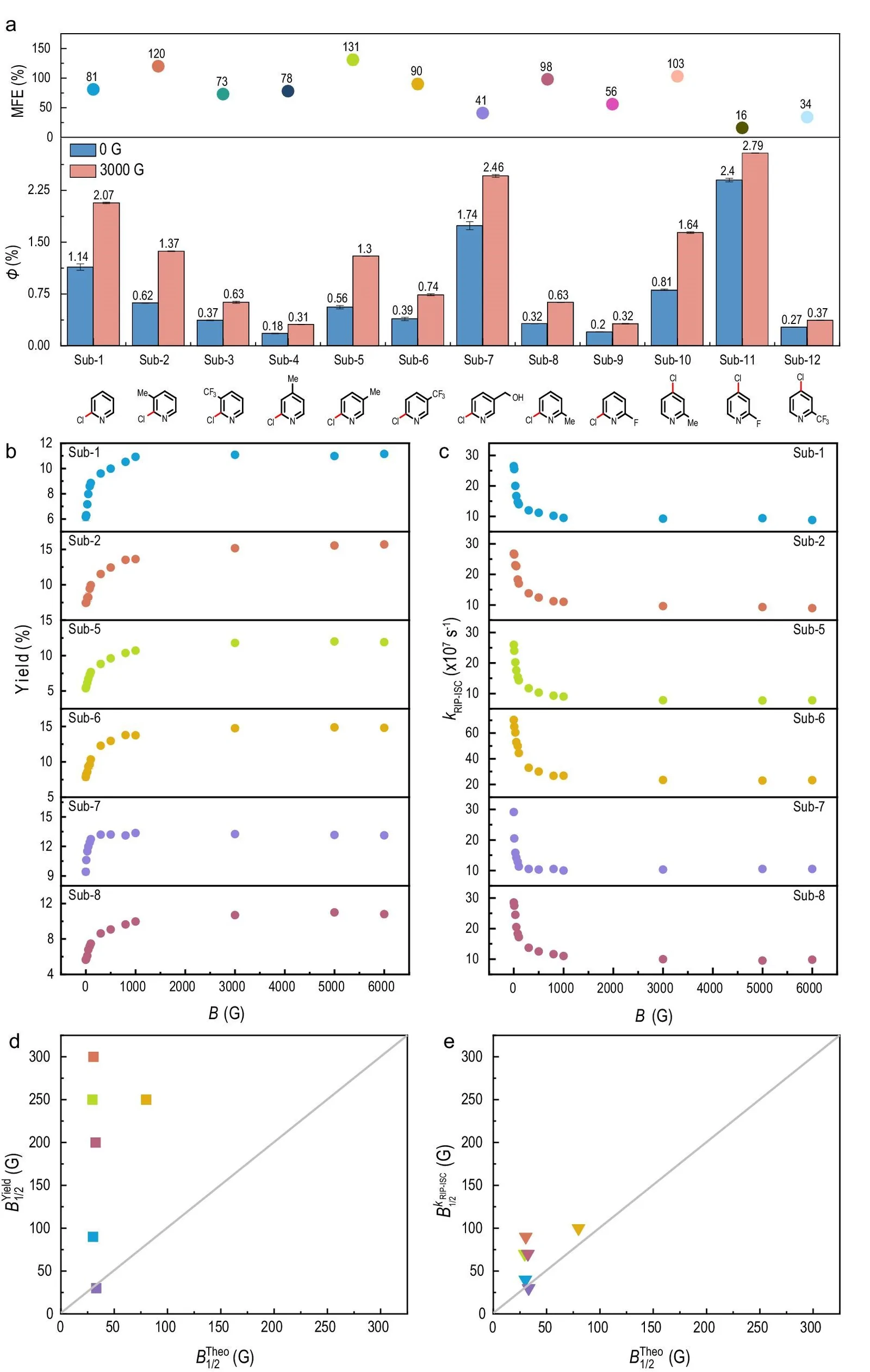
Substrate scope and validation of the kinetic model. (a) The MFE on the quantum yield (Φ) of the reaction (refer to ST-5 of the Supplementary data for the details) for a diverse range of chloropyridine substrates (Sub-1 to Sub-12) under a 3000 G magnetic field. MFE = [(Φ(B) − Φ(0))/Φ(0)] × 100%, where Φ(B) and Φ(0) represent the quantum yield in the presence and absence of an external magnetic field, respectively. (b) Magnetic field dependence of the reaction yield for a representative set of substrates. (c) Magnetic field dependence of the intrinsic spin interconversion rate (*k*_RIP-ISC_) for the selected substrates, deconvolved using the kinetic model. (d) Comparison of the half-saturation field derived from the yield ($B_{{\mathrm{1/2}}}^{{\mathrm{Yield}}}$) with the theoretical value ($B_{{\mathrm{1/2}}}^{{\mathrm{Theo}}}$) for selected substrates. (e) Comparison of the half-saturation field derived from the intrinsic rate ($B_{{\mathrm{1/2}}}^{{k}_{{\mathrm{RIP - ISC}}}}$) with the theoretical value ($B_{{\mathrm{1/2}}}^{{\mathrm{Theo}}}$) for selected substrates.

We further conducted transient spectroscopy studies on a representative set of substrates (Sub-1, Sub-2, Sub-5, Sub-6, Sub-7 and Sub-8; Figs S16–S20), which were selected to encompass a wide range of MFE values (40%–130%) and include both EDG and EWG. A consistent observation across all substrates investigated is that the cage-escape pathway of the ^1^CTC was invariably inefficient and unobservable. This finding corroborates our earlier assertion that low cage-escape efficiency is a hallmark characteristic of these chloropyridine-type substrates. However, quantitative analysis using our kinetic model reveals subtle but crucial differences. The model-derived cage-escape rate constants (Table S8) show that this process is even more suppressed for substrates bearing EDGs. Crucially, this kinetic insight provides direct validation for the observed substituent effect on the MFE, establishing that the modulation of cage-escape dynamics is the critical factor governing the magnitude of the MFE in this system.

To further validate our model, we systematically examined the variation of reaction yields with magnetic field strength for the selected substrates (Fig. 4b). For the vast majority of substrates, the MFE reached saturation at a relatively low magnetic field of ∼3000 G. This finding confirms the effective modulation of the reaction with modest magnetic field strength and supports a consistent HFCM across these different substrates. Notably, the half-saturation field derived from the magnetic field dependence curve of yield, $B_{{\mathrm{1/2}}}^{{\mathrm{Yield}}}$, exhibits a significant positive discrepancy from the theoretically predicted value, $B_{{\mathrm{1/2}}}^{{\mathrm{Theo}}}$, which can be visually reflected in the curve’s slow and reluctant approach to saturation. Applying our established kinetic model, we again deconvolved the data to extract the magnetic field dependence of the intrinsic spin interconversion rate, *k*_RIP-ISC_(*B*), for each substrate (refer to ST-4 of the Supplementary data for the details). As expected, *k*_RIP-ISC_ decreases with magnetic field strength according to the HFCM, exhibiting a trend opposite to that of the reaction yield, while simultaneously saturating more rapidly (Fig. 4c). Consistent with this behavior, the corresponding half-saturation field, $B_{{\mathrm{1/2}}}^{{k}_{{\mathrm{RIP - ISC}}}}$ is largely reduced in stark contrast to $B_{{\mathrm{1/2}}}^{{\mathrm{Yield}}}$, and shows better agreement with the theoretical value $B_{{\mathrm{1/2}}}^{{\mathrm{Theo}}}$ (Fig. 4d and e). These findings collectively provide robust support for the validity of our kinetic model, underscoring its essential role in correctly interpreting the intrinsic spin dynamics of this system.

### Magnetic field enhancement for practical photoredox transformation

Conventional investigations of the MFE in photochemistry and photoinduced electron transfer reactions have primarily focused on mechanistic understanding and typically been limited to the initial stages of the reaction [46,52,53]. In contrast, practical enhancements of photoredox reactions at high extents of conversion under low magnetic field strength remain largely unexplored, especially when applied across a broad range of substrates. Due to the complex mechanism underlying photoredox reactions, effective conditions observed at the initial stage of the reaction do not necessarily guarantee efficient results at high reaction extents [54]. Herein, we demonstrated the practical application of static magnetic field as a readily available spin-modulation strategy in enhancing photoredox transformation at high-conversion region. As shown in Fig. 5a, the photocatalytic yields of various substrates under both zero field and a 3000 G field were investigated. Notably, the application of an external magnetic field significantly enhances the reaction yields compared to the zero-field scenario, with the enhancement comparable to or even greater than that observed at low-conversion cases (Fig. 5b). Specifically, Sub-3 achieves an unprecedent yield enhancement of 160%. This observation unambiguously demonstrates the robustness of magnetic field enhancement strategy in our system. It should be noted that a magnetic field as low as 3000 G can be readily provided by permanent magnets, such as NdFeB, obviating the need for electromagnets or superconducting magnets, which would otherwise require additional substantial energy input to generate high magnetic fields.

**Figure 5.**
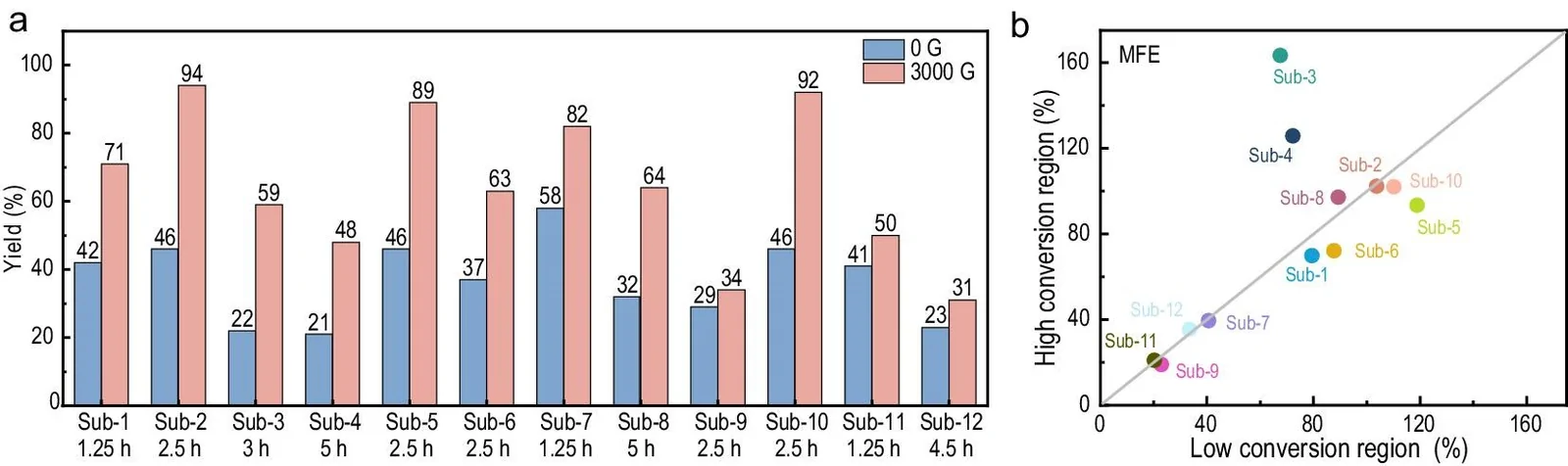
Practical application of magnetic field enhancement in photoredox transformations at high conversion. (a) Comparison of final reaction yields for various chloropyridine substrates at 0 and 3000 G. All reactions were conducted by irradiating with a CW laser (375 nm, 145 mW) at room temperature in 0.4 M SDS aqueous solution, containing 50 mM substrates, 5 mol% photocatalyst and 2.0 equivalents of DIPEA. (b) The MFE calculated from the reaction yields at high conversion and low conversion. MFE = [Yield(*B*) − Yield(0)]/Yield(0) × 100%, where Yield(*B*) and Yield(0) are the reaction yields in the presence and absence of a 3000 G magnetic field, respectively.

## CONCLUSIONS

In summary, this work establishes that a weak external magnetic field can serve as a remarkably potent and practical tool for enhancing photoredox catalysis, achieving an unprecedented yield enhancement of over 160%. We strategically targeted the hydrodechlorination of *N*-heteroaryl chlorides, a class of substrates notorious for their poor cage-escape efficiency. Our findings validate magnetic field modulation as a robust strategy for circumventing this limitation, demonstrating that spin control can effectively rescue reaction efficiency in systems dominated by tight RIPs. This mechanistic hypothesis is rigorously substantiated by both spectroscopic measurements and a novel inverse application of an established kinetic framework, which successfully decoupled intrinsic spin kinetics from convoluted reaction kinetics and resolved the discrepancy between theoretical and experimental half-saturation field values. By surpassing the critical efficiency-doubling threshold, this study decisively transitions magnetic field control from a physical curiosity into a robust synthetic tool and opens the door to a new paradigm in reaction design. We anticipate these findings will inspire the broader synthetic community to embrace spin modulation as a fundamental and powerful new handle for optimizing complex molecular transformations, paving the way for innovations in catalysis and molecular transformation.

## Supplementary Material

nwag450_Supplemental_File
