# Supplementary material for: Large low-field magnetic enhancement in photoredox hydrodechlorination of N-heteroarene chlorides restricted by poor cage escape
Source: Natl Sci Rev. 2026 Jul 24;13(19):nwag450. doi: 10.1093/nsr/nwag450 (PMC13715438; doi:10.1093/nsr/nwag450)
Supplement: Supplementary file 1 [file nwag450_supplemental_file.pdf]

Supporting Information

Large low-field magnetic enhancement in photoredox hydrodechlorination of *N*-heteroarene chlorides restricted by poor cage escape

Mingli Sun[1], Chenli Chen[2], Lingfang Chen[3], Shen Zhou[4], Jianyu Liu[4], Binghui Xue[5], Panchao Yin[5], Jiayi Liang[3], Shuming Bai[3,*], Jialong Jie[2,*], Hongmei Su[2], Song Gao[1,6,7] and Linan Zhou[1,*]

[1]Spin-X Institute, School of Chemistry and Chemical Engineering, Guangdong-Hong Kong-Macao Joint Laboratory of Optoelectronic and Magnetic Functional Materials, State Key Laboratory of Luminescent Materials and Devices, South China University of Technology, Guangzhou 511442, China;
[2]College of Chemistry, Beijing Normal University, Beijing 100875, China;
[3]Beijing National Laboratory for Molecular Sciences, State Key Laboratory for Structural Chemistry of Unstable and Stable Species, Institute of Chemistry, Chinese Academy of Sciences, Beijing 100190, China;
[4]College of Science, National University of Defense Technology, Changsha 410003 China;
[5]South China Advanced Institute for Soft Matter Science and Technology, Guangdong Basic Research Center of Excellence for Energy and Information Polymer Materials, State Key Laboratory of Luminescent Materials and Devices, South China University of Technology, Guangzhou 510641, China;
[6]Key Laboratory of Bioinorganic and Synthetic Chemistry of Ministry of Education, School of Chemistry, LIFM, IGCME, GBRCE for Functional Molecular Engineering, Sun Yat-Sen University, Guangzhou 510275, China and [7]Beijing National Laboratory of Molecular Science, Beijing Key Laboratory of Magnetoelectric Materials and Devices, College of Chemistry and Molecular Engineering, Peking University, Beijing 100871 China

***Corresponding authors.** E-mails: baishuming@iccas.ac.cn; jialong@bnu.edu.cn; zhoulinan@scut.edu.cn

Content

Materials and methods **3**

Materials 3

Photocatalytic measurements with external magnetic field 3

Small angle X-ray scattering 3

Viscosity Measurements 3

Time-resolved absorption spectroscopy 3

Nanosecond transient absorption 3

Femtosecond transient absorption 4

Spin-density distribution and hyperfine interactions constant of radical intermediates 4

Supplementary Text (ST) **5**

ST-1. Small angle X-ray scattering 5

ST-2. Theoretical simulation of magnetic field effect dependence on micelle size 7

ST-3. Global fitting of time-resolved absorption spectroscopy 9

ST-4. Kinetic model for determining k_{CE} and $k_{RIP\text{-}ISC}(B)$ 11

ST-5. Quantum yield measurements 14

Supplementary Figures and Tables **15**

References **43**

Materials and methods
Materials
Unless otherwise stated, all reagents and materials were obtained from commercial sources and used without further purification. Ultrapure water (resistivity > 18.2 MΩ·cm at 25 °C) was generated by a Clever-S Pure Water System(Shanghai Zhiang).

Photocatalytic measurements with external magnetic field
For a typical photocatalytic reaction, a 300 μL aliquot of a degassed aqueous solution of 0.4 M SDS, containing the substrate (1.0 equiv.), the photocatalyst (PC, 5 mol%), and DIPEA (2.0 equiv.), was placed in a reaction vessel. The mixture was irradiated with a 375 nm CW laser (145 mW; Model: MXL-III-375, Changchun New Industries) under an external magnetic field generated by an electromagnet (Model: WDS-50W, Shanghai Tindun). The zero-field condition (< 1.0 Gs) was established by nullifying the residual field with a countercurrent. To rigorously exclude any confounding influence from local heating or thermal convection induced by the electromagnet, the bulk solution temperature was measured immediately before and after the reaction under laser irradiation. The overall temperature increase of the reaction solution was strictly within 2.0 °C in both the absence (0 Gs) and presence (3000 Gs) of the magnetic field. This thermal stability confirms that the electromagnet does not induce any localized heating, and the observed yield enhancement is purely spin-chemical in origin. Detailed experimental parameters, including reagent amounts and irradiation times, are provided in **Table S7**. Upon completion, the reaction mixture was analyzed by high-performance liquid chromatography (HPLC) using a Shimadzu LC-20 system equipped with a ShimNex HE C18-Ph column (150 × 4.6 mm, 3 μm particle size). Analytes were separated using a mobile phase of acetonitrile and water, and their concentrations were quantified against calibration curves prepared from authentic standards.

Small angle X-ray scattering
Small-angle X-ray scattering (SAXS) data were acquired on a Xeuss 3.0 system (Xenocs SAS). The instrument is equipped with a D2+ MetalJet X-ray source (Excillum) generating Ga Kα radiation (wavelength, λ = 0.134 nm). All measurements were conducted at a sample-to-detector distance of 1600 mm, and the instrument was calibrated using a silver behenate standard prior to data collection.

Viscosity Measurements
The viscosity of different solvent systems and micellar solutions was measured using a rotational viscometer (Shanghai Xiniu Nabo Instrument Co., Ltd.). All measurements were performed at a constant room temperature of 25 °C. For each test, a 30 mL liquid sample was placed in a beaker and subjected to the measurement. The obtained viscosity values for the various systems are summarized in **Table S1**.

Time-resolved absorption spectroscopy
Nanosecond transient absorption
The nanosecond transient absorption (ns-TA) spectroscopy was performed on an Edinburgh LP980 spectrometer. The pump pulses were generated by the third harmonic (355 nm, fwhm ≈ 7 ns, 1 Hz) of a Q-switched Nd:YAG laser (Spectral Physics, Lab 170). A 150 W pulsed xenon lamp provided a broadband probe beam. For all experiments, samples were contained in a 1 cm path length quartz cuvette and maintained at ambient temperature (ca. 298 K). The probe light transmitted through the sample was directed into a monochromator and detected by a photomultiplier tube (PMT). Kinetic traces were

recorded from 300 to 700 nm using a digital oscilloscope (Tektronix, TDS 3012C, 1.25 Gs/s) and processed with the manufacturer's L900 software. Kinetic parameters were extracted from the data by fitting to appropriate models, with the goodness-of-fit judged by the weighted residuals and the reduced $\chi 2$ value.

Femtosecond transient absorption

Femtosecond transient absorption (fs-TA) spectroscopy was performed in a pump-probe configuration using a Harpia-TA spectrometer (Light Conversion) coupled with a Ti:Sapphire amplifier (Coherent Astrella; 40 fs, 800 nm, 1 kHz). The 355 nm pump pulses were generated by an optical parametric amplifier (OPA; TOPAS-C, Coherent) seeded by the 800 nm fundamental. A broadband white-light continuum (WLC) probe was produced by focusing a small fraction of the fundamental beam into a CaF_2 crystal. The temporal delay between the pump and probe pulses was controlled by a motorized delay stage. The pump beam was modulated at 500 Hz, enabling shot-to-shot referencing. To suppress rotational depolarization effects, the polarization of the pump was set at the magic angle (54.7°) relative to the probe. The two beams were made collinear and spatially overlapped within a 1 mm path length fused silica cuvette. The resulting two-dimensional data maps of differential absorbance (ΔA) as a function of wavelength and time delay were subjected to global analysis using the Glotaran software. All experiments were conducted at ambient temperature (ca. 298 K).

Spin-density distribution and hyperfine interactions constant of radical intermediates

Density functional theory (DFT) calculations were performed with the Gaussian16 program suite (Revision A.03)[1]. All calculations on these open-shell systems utilized the UB3LYP functional[2]. First, the geometries of the PC radical cation and substrate radical anions were fully optimized at the UB3LYP/def2-TZVP level of theory (see **Table S9** for coordinates). Harmonic frequency analysis at this same level confirmed each stationary point as a true local minimum (zero imaginary frequencies). With the optimized geometries, isotropic hyperfine coupling constants (a_{iso}) were then computed at the UB3LYP/pcH-2 level. The calculated spin densities at each nucleus N, $\rho(N)$, were converted to a_iso values (in Gauss) via the relation cited[3]: $a_{\mathrm{iso}}(N) = 142.770\, \langle S_z \rangle^{-1} g_N \rho(N)$. For visualization, spin density distributions were rendered as isosurfaces in VMD[4] and as two-dimensional contour maps using Python's Matplotlib library.

Supplementary Text (ST)

ST-1. Small angle X-ray scattering

The SasView software was used to fit the experimental data, and the details of fitting models used are listed below.

For SDS solutions with substrates, two kinds of combined models were used.

For concentrations ranging from 0.01 M to 0.1 M, the SAXS data were fitted using sphere model combined with power law[5] and a structure factor of Hayter_msa[6-9]. The scattering function of the combined model was manipulated as follows:

$$\text{Combined\_model} = \text{scale\_factor1} \times (\text{scale\_factor2} \times \text{sphere\_model} \times \text{Hayter\_msa} + \text{scale\_factor3} \times \text{power\_law}) + \text{background}$$

The crucial parameters are listed below, radius-effective corresponds to the effective size obtained from the structure factor, while radius is the size obtained from the form factor of sphere (**Figure S4** and **Table S2**).

For concentration ranging from 0.2 M to 0.8 M, the SAXS data were fitted using core shell ellipsoid model[6, 10] combined with power law and a structure factor of Hayter_msa. The scattering function of the combined model was manipulated as follows:

$$\text{Combined\_model} = \text{scale\_factor1} \times (\text{scale\_factor2} \times \text{core\_shell\_ellipsoid\_model} \times \text{Hayter\_msa} + \text{scale\_factor3} \times \text{power\_law}) + \text{background}$$

The crucial parameters are listed below, radius-effective corresponds to the effective size obtained from the structure factor. Radius-core-equator and radius-core-polar correspond to the radius of core in the equatorial direction and polar/meridian direction. Shell-thickness-equator and shell-thickness-polar are the shell thickness of core shell ellipsoid in the equatorial direction and polar/meridian direction (**Figure S5** and **Table S3**).

For solutions of other surfactants, different combined models were used.

For K-EL, Tween-80, Brij-35 with substrates, core shell sphere model[5] together with structure factor of hard sphere[6, 11] should be used considering the nonionic nature of this surfactant:

$$\text{Combined\_model} = \text{scale\_factor1} \times (\text{scale\_factor2} \times \text{core\_shell\_sphere\_model} \times \text{hardsphere} + \text{scale\_factor3} \times \text{power\_law}) + \text{background}$$

The crucial parameters are listed below, radius-effective corresponds to the effective size obtained from the structure factor. Radius-core and shell-thickness correspond to the size parameters of the form factor of core shell sphere(**Figure S6** and **Table S4**).

The TX-100 system is different from the above, as it can be well fitted using single sphere model, while the successful fitting of TX-100 with substrates requires combined model:

$$\text{Combined\_model} = \text{scale\_factor1} \times (\text{scale\_factor2} \times \text{core\_shell\_sphere\_model} \times \text{hardsphere} + \text{scale\_factor3} \times \text{power\_law}) + \text{background}$$

The crucial parameters are listed below, radius-effective corresponds to the effective size obtained from the structure factor. Radius-core and shell-thickness correspond to the size parameters of the form factor of core shell sphere. For TX-100, we can only obtain the radius exported from the fitting of sphere model(**Figure S7** and **Table S5**).

For CTAC with substrates, the combined model is similar to the model used for SDS system, while the we used form factor of sphere here:

$$\text{Combined\_model} = \text{scale\_factor1} \times (\text{scale\_factor2} \times \text{sphere\_model} \times \text{Hayter\_msa} + \text{scale\_factor3} \times$$

power_law) + background

The crucial parameters are listed below, radius-effective corresponds to the effective size obtained from the structure factor, while radius is the size obtained from the form factor of sphere(**Figure S8** and **Table S6**).

ST-2. Theoretical simulation of magnetic field effect dependence on micelle size

(1) Theoretical framework and basic assumptions

To quantitatively elucidate the experimentally observed dependence of the MFE on micelle sizes, we have developed a theoretical model. The core mechanism of this model is based on the competition between the spin-exchange interaction and the hyperfine interaction[12]. It directly correlates the strength of the spin-exchange interaction with the spatial distribution of RIPs confined within the micellar core, thereby revealing the evolution of the MFE as a function of micelle size.

In a spin-correlated radical ion pair system, the MFE is primarily governed by two key parameters:

- Spin-Exchange Interaction ($2J$): Characterizes the strength of the electron exchange coupling between the two spin centers.
- Effective Hyperfine Interaction (a_{eff}): Describes the effective strength of the coupling between the electron spin and the surrounding nuclear spins.

The relative magnitudes of these two parameters determine the magnetic response behavior of the system. When the exchange interaction is much larger than the hyperfine interaction ($|2J| \gg a_{\mathrm{eff}}$), the system is in the exchange-dominated regime. In this regime, the energy level splitting between the singlet (S) and triplet (T) states is significant, and S-T spin-mixing is effectively suppressed. Consequently, the spin conversion between the radical ions becomes nearly unresponsive to an external magnetic field, and the MFE approaches zero. Conversely, when the magnitude of J is comparable to a_{eff} ($|2J| \approx a_{\mathrm{eff}}$), ISC between the S and T states becomes efficient, and the system exhibits high sensitivity to an external magnetic field[12]. Within this regime, however, a further increase in inter-radical distance and the corresponding decrease in spin exchange interaction will shorten the lifetime of the germinate RIP, which in turn diminishes the system's magnetic sensitivity.

The spin-exchange interaction can be described by the Hamiltonian[13]:

$$H_{\mathrm{ex}} = -2J\mathbf{S}_1 \cdot \mathbf{S}_2$$

where $\mathbf{S}_1$and $\mathbf{S}_2$ are the spin operators of the two radicals, and J is the exchange coupling constant. The value of J is extremely sensitive to the inter-radical distance, *r*. In the weak coupling limit, this dependence can be approximated by a power-law decay[14]:

$$J(r) \propto r^{-6}$$

Based on this, we introduce a cut-off distance (r_{c}) as a threshold to distinguish between different magnetic response regimes (**Figure S9a**):

- When $r < r_{\mathrm{c}}$, the exchange interaction is dominant, placing the system in a magnetically silent regime where the MFE is completely suppressed.
- When $r \geq r_{\mathrm{c}}$, the system enters a magnetically sensitive regime. The strength of its magnetic field response diminishes as *r* increases, following the relation:

$$\mathrm{MFE} \propto J(r) \propto r^{-6}$$

(2) Geometric distribution model for intra-micellar RIP separation

The model assumes that the spin-bearing radical ions can be treated as independent point particles uniformly distributed within a spherical micelle of radius *R* (Note: SDS micelles at 0.2-0.8 M are

ellipsoidal; here, we use their volume-equivalent spherical radius). According to geometric probability theory[15], the Probability Density Function (PDF), $P(r)$, for the distance r between two random points within a sphere is given by:

$$P(r) = \frac{3r^2}{R^3} - \frac{9r^3}{4R^4} + \frac{3r^5}{16R^6}, 0 < r < 2R$$

However, due to constraints imposed by finite volume, steric hindrance, and electrostatic interactions, we define physical boundaries for the inter-spin distance r:

- Lower Bound (r_{lb}): The closest contact distance, determined by the sum of the van der Waals distance between two molecules, so $r_{\mathrm{lb}} = \mathrm{d}$，In this study, it is estimated to be 0.71 nm[16].
- Upper Bound (r_{ub}): The maximum possible separation for two radicals coexisting within the same micelle, approximated as $r_{\mathrm{ub}} = 2R - \mathrm{d}$.

For proper normalization, $P(r)$ must be normalized over the physically allowed interval $[r_{\mathrm{lb}}, r_{\mathrm{ub}}]$:

$$\int_{r_{lb}}^{r_{ub}} P(r)\, dr = 1$$

Subsequently, the average radical ion pair separation, $\bar{r}$, can be calculated for different micelle sizes, yielding the dependence of $\bar{r}$ on the micelle diameter D, and the van der Waals distance (**Figure S9b**).

(3) Calculation of MFE and model parameter optimization

Integrating the principles outlined above, the overall MFE for a micelle of a given radius R can be obtained by performing an ensemble average over all possible radical pair separations:

$$\mathrm{MFE} = \int_{r_{\mathrm{lb}}}^{r_{\mathrm{ub}}} \mathrm{MFE}\,(r) P(r) dr$$

Considering the effect of the cut-off distance r_{c}, where the system only contributes to the MFE when $r \geq r_{\mathrm{c}}$, the above equation can be specified as:

$$\mathrm{MFE} = A \int_{r_{\mathrm{c}}}^{r_{\mathrm{ub}}} r^{-6}\, P(r) dr$$

where A is a proportionality constant that incorporates the hyperfine coupling constant and other experimental parameters.

By employing a least-squares fitting method, the dependence of MFE on micelle diameter can be determined for different r_c (**Figure S9c**). A comparison with experimental data yields an optimal cut-off distance, r_{c}, of 1.73 nm.

ST-3. Global fitting of time-resolved absorption spectroscopy

(1) Determination of the extinction coefficient of ^{3}PC*

The extinction coefficient of the triplet excited state of the photocatalyst (^{3}PC*) was determined using nanosecond transient absorption (ns-TA) spectroscopy via the triplet-triplet energy transfer (TTET) method[17]. 1,1-Binaphthyl (BN) was employed as the triplet energy acceptor, for which the extinction coefficient at 615 nm is known (ε_{615nm} = 11300 dm^3·mol^{-1}·cm^{-1}).

The intrinsic decay of ^{3}PC* was monitored at its characteristic absorption maximum at 460 nm, yielding a lifetime (τ_0) of 20 μs and a first-order rate constant (k_0) of 5.0×10^4 s^{-1}. The TTET process was subsequently investigated by observing the quenching of the ^{3}PC* signal at 460 nm and the concomitant formation of the ^{3}BN* signal at 615 nm upon addition of BN. The observed decay rate constant of ^{3}PC* (k_t) was determined at various concentrations of BN. A linear fit of the plot of k_t versus [BN] afforded the second-order rate constant for energy transfer (k_q) between ^{3}PC* and BN, which was found to be 3.7×10^9 M^{-1} s^{-1}.

$$PC \xrightarrow{h\nu,\ ISC} {}^3PC^* \qquad k_t = k_0 + k_q[BN]$$

$$^3PC^* \xrightarrow{k_0} PC \qquad ratio = k_q[BN]/k_t$$

$$^3BN^* = ratio \times {}^3PC^*$$

$$^3PC^* + BN \xrightarrow{k_q[BN]} {}^3BN^* + PC \qquad \Delta OD_{615nm}/\varepsilon_{615nm} = ratio \times \Delta OD_{460nm}/\varepsilon_{460nm}$$

The maximum concentration of ^{3}BN* generated upon complete energy transfer was calculated from the maximum absorbance change at 615 nm using its known extinction coefficient. Based on the stoichiometry of the energy transfer process, this concentration corresponds to the initial concentration of ^{3}PC* that was quenched. By correlating this concentration with the initial absorbance change of ^{3}PC* at 460 nm (optical path length = 1 cm), the molar extinction coefficient of ^{3}PC* at 460 nm was calculated to be 17500 dm^3·mol^{-1}·cm^{-1}.

(2) Determination of the quantum yield of ^{3}PC*

The triplet quantum yield of PC (Φ_T) was determined using a relative method with benzophenone (BP) as the actinometer, which has a known triplet quantum yield of unity (Φ_T = 1) and a triplet extinction coefficient of 7100 dm^3·mol^{-1}·cm^{-1} at 520 nm[18]. Solutions of PC and BP were prepared to have identical absorbance at the laser excitation wavelength of 355 nm.

Under identical excitation conditions (355 nm laser pulse), the transient absorption spectra of both samples were recorded. The maximum concentration of the generated triplet state for each species ($[^3PC^*]_{max}$ and $[^3BP^*]_{max}$) was determined from the initial maximum absorbance change (ΔOD_{max}) in their respective kinetic traces and their known extinction coefficients, according to the Beer-Lambert law. Specifically, $[^3PC^*]_{max}$ was calculated from the ΔOD_{max} at 460 nm using the extinction coefficient determined in the previous step (ε_{460nm} = 17500 dm^3·mol^{-1}·cm^{-1}), while $[^3BP^*]_{max}$ was calculated from the ΔOD_{max} at 520 nm using its literature value (ε_{520nm} = 7100 dm^3·mol^{-1}·cm^{-1}).

The quantum yield of ^{3}PC* was then calculated from the ratio of the maximum triplet concentrations, as described by the following equation:

$$\Phi_T(PC)/\Phi_T(BP) = [^3PC^*]_{max}/[^3BP^*]_{max}$$

This analysis yielded a triplet quantum yield (Φ_T) for PC of 0.87, a value consistent with those reported in the literature for similar photocatalysts[19-21].

(3) Determination of the extinction coefficient and quantum yield of ^{1}PC*

With the extinction coefficient and quantum yield of ^{3}PC* established, the extinction coefficient of the

singlet excited state (^{1}PC*) at 650 nm was derived from the femtosecond transient absorption (fs-TA) data of PC. The calculation utilized the following equation, which relates the initial singlet absorption to the subsequent triplet absorption signal:

$$\varepsilon_{650nm}(^1PC^*) = [\Phi_T(^3PC^*)*\triangle OD_{max}(650nm)*\ \varepsilon_{460nm}(^3PC^*)]/\triangle OD_{max}(460nm)$$

Using this method, the extinction coefficient for ^{1}PC* at 650 nm was determined to be 20700 $dm^3 \cdot mol^{-1} \cdot cm^{-1}$.

(4) Determining the branching ratios and rate constants of the three decay pathway for CTC

The photophysically determined extinction coefficients were subsequently used to quantify the species involved in the decay of the CTC. The maximum concentration of the initially formed singlet excited state, $[^1PC^*]_{max}$, was determined from the initial absorbance at 650 nm in the fs-TA experiment and its newly calculated extinction coefficient. The concentration of ^{3}PC* generated from CTC decay, $[^3PC^*]_{CTC}$, was determined from the amplitude of the ^{3}PC* signal in the transient spectrum at a long delay time (t = 7 ns) and its extinction coefficient. Similarly, the concentration of the free photocatalyst radical cation ($PC^{\bullet+}$) formed from CTC decay, $[PC^{\bullet+}]_{CTC}$, was determined from its spectral contribution at t = 7 ns and its respective extinction coefficient.

The reference spectrum and extinction coefficient for $PC^{\bullet+}$ were characterized independently. Nanosecond transient absorption spectra of PC under aerobic conditions were recorded following 355 nm laser excitation. Although ^{3}PC* exhibits a long lifetime under anaerobic conditions (~tens of microseconds), it is rapidly quenched by dissolved O_2 in aerobic solution (~tens of nanoseconds). This quenching can proceed via an electron transfer pathway ($^3PC^* + O_2 \rightarrow PC^{\bullet+} + O_2^{\bullet-}$), leading to the formation of $PC^{\bullet+}$. Consequently, the ns-TA spectra recorded under these conditions are dominated by the characteristic absorption of $PC^{\bullet+}$, consistent with previously reported data[22, 23]. This allowed for the reliable quantification of $[PC^{\bullet+}]_{CTC}$.

Assuming that the interaction of ^{1}PC* with a large excess of substrate (1 M) leads to the exciplex, which then converts to the CTC with 100% efficiency, the branching ratios for the formation of ^{3}PC* and free $PC^{\bullet+}$ from the CTC can be calculated as $[^3PC^*]_{CTC}/[^1PC^*]_{max}$ and $[PC^{\bullet+}]_{CTC}/[^1PC^*]_{max}$, respectively. The branching ratio of the non-productive back electron transfer (BET) pathway, which returns the radical ion pair (RIP) to the ground state, is then calculated by subtracting the other two ratios from unity:

$$1 - \frac{[^3PC^*]_{CTC}}{[^1PC^*]_{max}} - \frac{[^3PC^{\bullet+}]_{CTC}}{[^1PC^*]_{max}}$$

Once the branching ratios for the three decay pathways were established, their individual rate constants were calculated by partitioning the overall decay rate constant of the CTC (obtained from its lifetime) according to these ratios.

ST-4. Kinetic model for determining k_{CE} and $k_{RIP\text{-}ISC}(B)$

According the establish kinetic model we proposed in the main text, the magnetic field effect of product yield ($MFE_{Yield}(B)$) depends on multiple elementary kinetic constants, including the cage escape rate (k_{CE}), the back electron transfer rate (k_{BET}), the triplet charge recombination rate (k_{TCR}), and the field-dependent RIP intersystem crossing rate ($k_{RIP\text{-}ISC}(B)$). Mathematically, the $MFE_{Yield}(B)$ is a function of the four rate constants. Thus, in principle, any rate constant can be expressed as function of MFE and the other rate constant. However, the resulted function expressions are generally too complicated, so that we obtain the unknown rate constant by adjusting it with the other three known constant from spectroscopy measurement to best fit the $MFE_{Yield}(B)$.

A $MFE_{Yield}(B)$ can be interpreted as the relative difference in the valid probability of a photoredox cycle under a given magnetic field ($P(B)$) compared to that at zero field ($P(0)$), as described by Equation (1).

$$MFE_{Yield}(B) = \frac{P(B) - P(0)}{P(0)} \times 100\% \quad (1)$$

$P(B)$ is the sum of the probabilities of radical formation from ^{1}CTC, $P^S(B)$, and ^{3}CTC, $P^T(B)$, respectively, under a given magnetic field. These probabilities further depend on the distribution of decay pathways for ^{1}CTC and ^{3}CTC, described as follows.

Moreover, within the nonpolar confined environment of micelles, the Gibbs free energy of CTC and RIP is postulated to remain close due to competing positive enthalpy and entropy changes associated with charged particle separation in a weakly electrostatically screened medium. This results in the instantaneous establishment of equilibrium between CTC and RIP for both singlet and triplet manifolds. Therefore, these species can form effective recycle, with ineffective BET process from ^{1}CTC and effective free - radical formation from both ^{1}CTC and ^{3}CTC as the exits.

It should be noted that ^{1}CTC exhibits three decay pathways, corresponding to cage escape (CE), back electron transfer (BET), and triplet charge recombination (TCR) respectively. The branching ratios of these pathways satisfy:

$$f_{CE} + f_{BET} + f_{TCR} = 1 \quad (2)$$

among them, the specific expression for f_{CE} :

$$f_{CE} = \frac{k_{CE}}{k_{QCTC}} = \frac{k_{CE}}{k_{CE} + k_{BET} + k_{TCR}} \quad (3)$$

Here, k_{CE}, k_{BET}, k_{TCR} represent the rate constants of cage escape, back electron transfer, and triplet charge recombination respectively, and $k_{CE} + k_{BET} + k_{TCR}$ denotes the total decay rate of ^{1}CTC (k_{QCTC}). It is worth mentioning that the rate of the cage escape (CE) step is extremely slow, making it undetectable within the detection limit of the instrument.

When the ^{1}CTC was initially generated from chemical quenching of ^{1}PC* by the substrate, a portion with a proportion of f_{CE} will form free radicals that are effective for the photoredox reaction, while another portion with a proportion of f_{TCR} undergoes the TCR process to generate ^{3}PC*, both of which were derived from global analysis of the transient spectra (see **ST-3** for detailed method). According to triplet - state kinetics, ^{3}PC* will be almost completely quenched by the substrate in SDS micelle to generate ^{3}CTC (efficiency > 99%), part of which leads to regeneration of ^{1}CTC through the RIP-ISC process with a proportion of $f_{RIP-ISC}(B)$ (Equation 4), which further depends on the external magnetic field.

$$f_{RIP-ISC}(B) = \frac{k_{RIP-ISC}(B)}{k_{RIP-ISC}(B) + k_{CE}} \quad (4)$$

Thus, over a cycle, a proportion of $f_{TCR} * f_{RIP-ISC}(B)$ of ^{1}CTC will be regenerated, which again

contributes to free radical formation by a proportion of $f_{CE} * f_{TCR} * f_{RIP-ISC}(B)$. By analogy, for the *i*-th recycle, the proportion of free radical formation from ^{1}CTC will be $f_{CE}\left(f_{TCR} * f_{RIP-ISC}(B)\right)^i$ (note: the initially formed ^{1}CTC can be viewed as the zeroth recycle). Thus, the total probability of radical formation from singlet path can be viewed as the sum of a geometric series with the first term of f_{CE} and the common ratio of $f_{TCR} * f_{RIP-ISC}(B)$:

$$P^S(B) = \sum_{i=0}^{n-1} f_{CE}\left(f_{TCR} * f_{RIP-ISC}(B)\right)^i = \frac{f_{CE} * \left(1 - \left(f_{TCR} * f_{RIP-ISC}(B)\right)^n\right)}{1 - f_{TCR} * f_{RIP-ISC}(B)} \quad (5)$$

With large enough recycle times,

$$P^S(B) \cong \frac{f_{CE}}{1 - f_{TCR} * f_{RIP-ISC}(B)} \quad (6)$$

as $f_{TCR} * f_{RIP-ISC}(B) < 1$.

Similarly, the probability of radical formation from ^{3}CTC at the first run (the zeroth recycle) is $f_{TCR} * (1 - f_{RIP-ISC}(B))$, as ^{3}CTC was first generated with a probability of f_{TCR}, followed by the diffusion probability of $(1 - f_{RIP-ISC}(B))$ to form the free radical. As mentioned above, a proportion of $f_{RIP-ISC}(B)$ of ^{3}CTC will lead to ^{1}CTC through the RIP - ISC process, which can recycle back to ^{3}CTC with a probability of f_{TCR} via TCR process again. Thus, over a cycle, a proportion of $f_{TCR} * f_{RIP-ISC}(B)$ of ^{3}CTC is also regenerated. For the *i*-th recycle, the proportion of free radical formation from ^{3}CTC will be $f_{TCR} * (1 - f_{RIP-ISC}(B))\left(f_{TCR} * f_{RIP-ISC}(B)\right)^i$. Thus, the total probability of radical formation from the triplet path can be viewed as the sum of a geometric series with the first term of $f_{TCR} * (1 - f_{RIP-ISC}(B))$ and the common ratio of $f_{TCR} * f_{RIP-ISC}(B)$:

$$P^T(B) = \frac{f_{TCR} * (1 - f_{RIP-ISC}(B)) * \left(1 - \left(f_{TCR} * f_{RIP-ISC}(B)\right)^n\right)}{1 - f_{TCR} * f_{RIP-ISC}(B)} \quad (7)$$

Again, with large enough recycle times,

$$P^T(B) \cong \frac{f_{TCR} * \left(1 - f_{RIP-ISC}(B)\right)}{1 - f_{TCR} * f_{RIP-ISC}(B)} \quad (8)$$

Overall,

$$P(B) = P^S(B) + P^T(B) = \frac{f_{CE} + f_{TCR} * (1 - f_{RIP-ISC}(B))}{1 - f_{TCR} * f_{RIP-ISC}(B)} \quad (9)$$

According to Equations 4 and 9, the magnetic field effect is ultimately attributed to the magnetic field dependence of the hyperfine coupling-driven intersystem crossing rate of RIPs, $k_{RIP-ISC}(B)$.

The value of $k_{RIP-ISC}(B)$ at zero field, $k_{RIP-ISC}(0)$, can be estimated using an empirical formulate (Equation 10)[24], which is related to the half-saturation field of the RIP.

$$k_{RIP-ISC}(0) = \frac{1}{2}\frac{g\mu_B B_{1/2}^{\text{Theo}}}{\hbar} \quad (10)$$

The half-saturation field $B_{1/2}$ is a key parameter that characterizes the magnetic sensitivity of the intersystem crossing process. Its theoretical value $B_{1/2}^{\text{Theo}}$ can be estimated using an empirical formula, as shown below in Equation (11)[25]:

$$B_{1/2}^{\text{Theo}} = 2\frac{B_{PC^{\cdot+}}^2 + B_{Sub^{\cdot-}}^2}{B_{PC^{\cdot+}} + B_{Sub^{\cdot-}}} \quad (11)$$

Here, $B_{PC^{\cdot+}}$ and $B_{Sub^{\cdot-}}$ denote the effective hyperfine coupling strengths of the photocatalyst radical cation ($PC^{\bullet+}$) and the substrate radical anion (Sub-$1^{\bullet-}$), respectively. These quantities are derived from

the hyperfine coupling constants (A_{ik}) and nuclear spin quantum numbers (I_k) of all nuclei within each radical, as described by Weller Equation (12)[25]:

$$B_i = \sqrt{\sum_k A_{ik}^2 I_k(I_k+1)} \quad i = PC^{\cdot+}, Sub^{\cdot-} \quad (12)$$

The spin density distributions of $PC^{\bullet+}$ and Sub-$1^{\bullet-}$ can be obtained from DFT calculations (**Table S9**), allowing the determination of all hyperfine coupling constants for each nucleus within both radicals (**Figures S21 and S22**). This establishes a theoretical connection between microscopic nuclear parameters and the macroscopic manifestation of the magnetic field effect.

Moreover, by assuming equal rates for ISC between the three sublevels of the triplet state and the singlet state at zero field, the ISC rate under magnetic fields beyond the saturated field, $k_{RIP-ISC}(B \geq B_{\text{sat}})$, can be set to one-third of its zero-field value (Equation 13)[26].

$$k_{RIP-ISC}(B \geq B_{\text{sat}}) = \frac{1}{3} k_{RIP-ISC}(0) \quad (13)$$

With this framework, we can deconvolve the intrinsic spin dynamics from the overall reaction kinetics. The rate constants k_{BET} and k_{TCR}, and thus the branching ratio f_{TCR}, were determined directly from the global analysis of fs-TA data (**Table S8**). The experimentally measured saturated MFE, $\text{MFE}_{\text{Yield}}(B_{\text{sat}})$,. The spin at zero field and saturated field were estimated from Weller's equation as described above. Therefore, the value of k_{CE} is then determined by adjusting it as the sole unknown parameter until the MFE function expression reproduces the experimentally measured $\text{MFE}_{\text{Yield}}(B_{\text{sat}})$.

With k_{CE} thus determined, the now fully parameterized MFE function expression allows for the back-calculation of the intrinsic ISC rate, $k_{\text{RIP-ISC}}(B)$, from any given experimental $\text{MFE}_{\text{Yield}}(B)$ measured at magnetic fields other than the saturated field. By plotting the calculated $k_{\text{RIP-ISC}}(B)$ against B, we can determine the intrinsic half-saturation field, $B_{1/2}^{k_{RIP-ISC}}$. This value represents the true magnetic sensitivity of the spin conversion process, free from the convolution of other kinetic steps, and can be directly compared with the theoretically predicted $B_{1/2}^{\text{Yield}}$ to validate our model. The parameters derived from this comprehensive analysis for all substrates are compiled in **Table S8**.

ST-5. Quantum yield measurements

The efficiency of the photoreaction was quantified by determining its quantum yield (Φ), defined as the number of product molecules generated per photon absorbed[27]. The value of Φ was determined according to Equation 14:

$$\Phi = \frac{Number\ of\ Molecules\ Formed}{Number\ of\ Photons\ Absorbed} = \frac{Y * c_{prod} * V * N_A}{\frac{\int_0^t P_{inc} - P_{trans}\, dt}{\frac{hc}{\lambda}}} \quad (14)$$

Here, the number of formed product molecules was calculated from the concentration of the product (c_{prod}) measured in the sample volume (V), accounting for the chemical yield (Y), with N_A as Avogadro's constant. The total number of absorbed photons was determined by continuously monitoring the incident (P_{inc}) and transmitted (P_{trans}) power of the monochromatic light source (wavelength λ) through the sample. The time-integral of the power difference yields the total absorbed energy, which was then divided by the energy per photon (hc/λ) to give the total photon count. The terms h and c represent Planck's constant and the speed of light, respectively.

Supplementary Figures and Tables

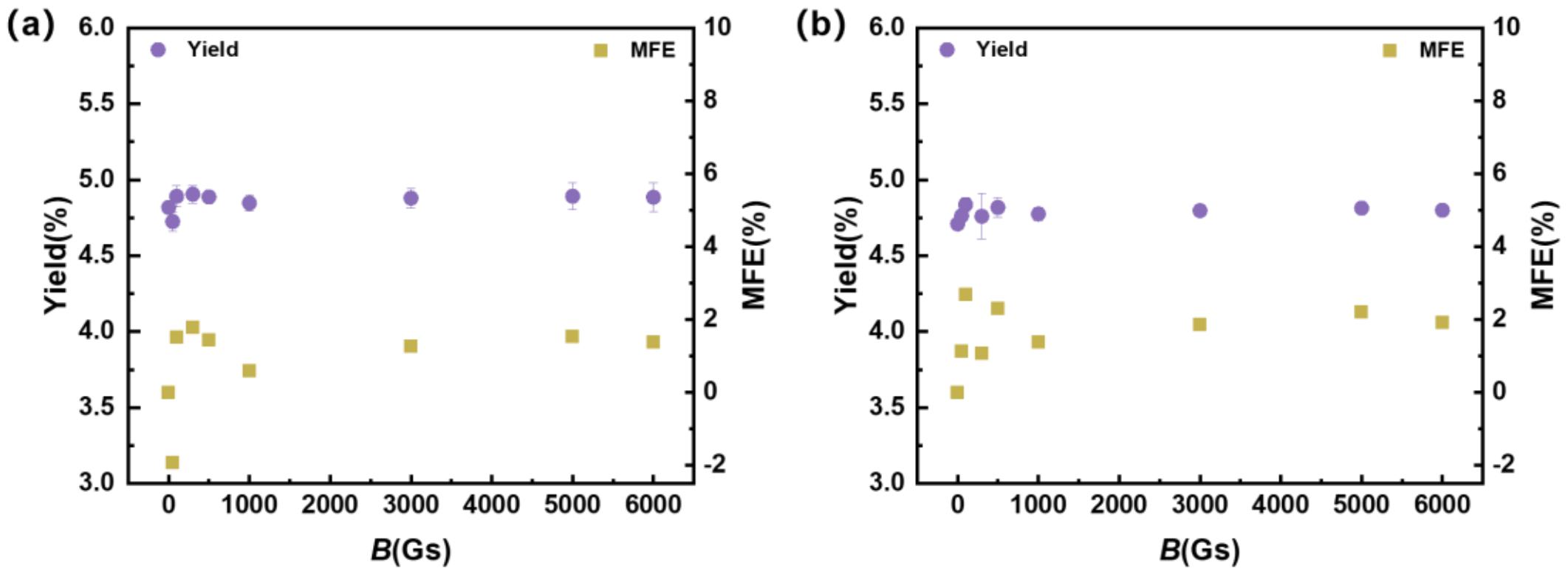

Figure S1: The field-dependent trends of yield and magnetic field effects in different reaction media: (**a**) PEG-400:H_2O (V:V=3:8) and (**b**) ILs:DMSO (V:V=1:19). Both media have a viscosity of ~2.4 cP, which is identical to that of 0.4 M SDS. The magnetic field effect is defined as: MFE = [(Yield(B) − Yield(0))/Yield(0)] × 100%, where Yield(B) and Yield(0) represent the product yields in the presence and absence of an external magnetic field, respectively.

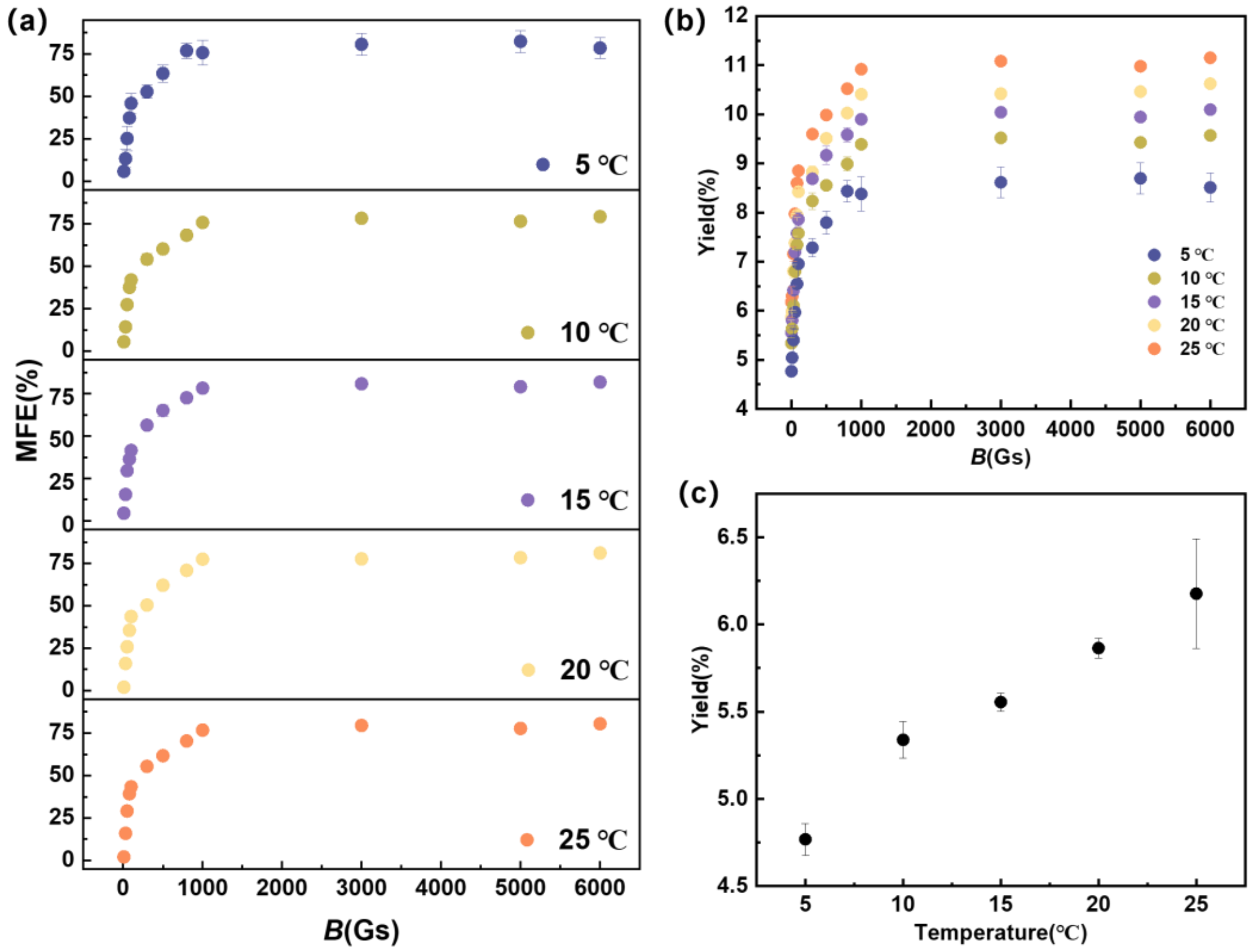

Figure S2: Temperature-dependent experiments for the representative substrate Sub-1. (**a**) The field-dependent trend of magnetic field effects (MFE) at different temperatures (5 ℃ to 25 ℃). (**b**) The field-dependent trend of product yield at different temperatures. (**c**) The dependence of the zero-field product yield on temperature. The magnetic field effect is defined as: MFE = [(Yield(B) − Yield(0))/Yield(0)] × 100%, where Yield(B) and Yield(0) represent the product yields in the presence and absence of an external magnetic field, respectively.

Note: As illustrated in **Figure S2**, while the zero-field product yield exhibits a typical positive temperature dependence (**Figure S2c**), the corresponding MFEs remain remarkably constant, plateauing at approximately 75–80% across the entire 5–25 ℃ range (**Figure S2a**). This stark contrast unambiguously demonstrates that the strong micellar cage effect is highly robust within this temperature window. Furthermore, it effectively rules out the possibility that temperature-induced macroscopic variations (such as decreased bulk viscosity or enhanced thermal diffusion) dictate the MFE, thereby reinforcing the conclusion that the unique micellar microenvironment plays the dominant role in generating the prominent MFEs.

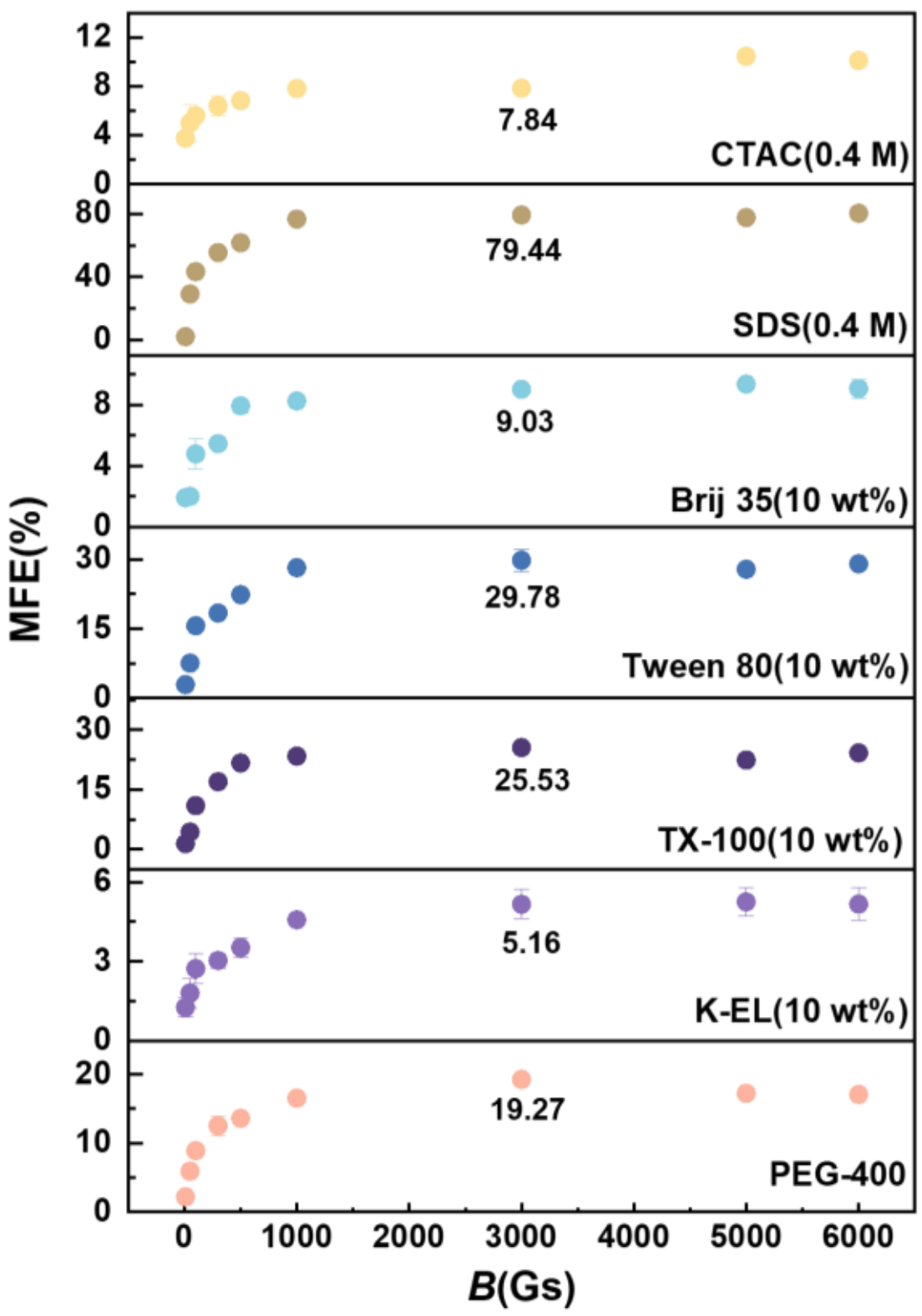

Figure S3: The field-dependent trend of magnetic field effects in different reaction media. The magnetic field effect is defined as: MFE = [(Rate(B) − Rate(0))/Rate(0)] × 100%, where Rate(B) and Rate(0) represent the initial reaction rates in the presence and absence of an external magnetic field, respectively.

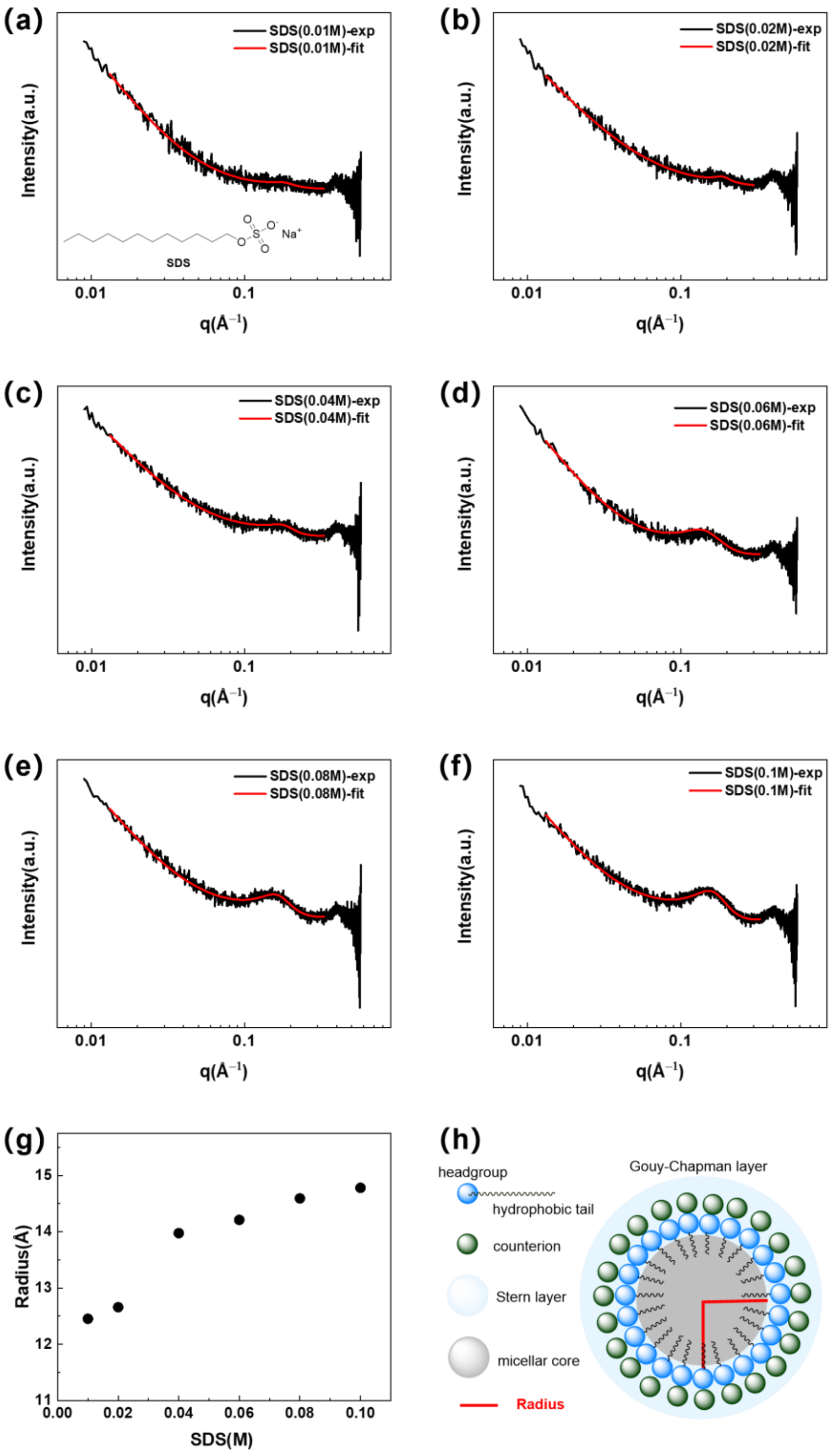

Figure S4: SAXS analysis of SDS micellar solutions (0.01–0.1 M) containing 50 mM Sub-1, 2.5 mM PC, and 100 mM DIPEA. **(a-f)** Experimental SAXS data (black line) and corresponding model fits (red line) for each SDS concentration. **(g)** Plot showing the micelle core radius as a function of SDS concentration. **(h)** Schematic illustration of the spherical micelle model used for fitting. The inset provides a structure of SDS.

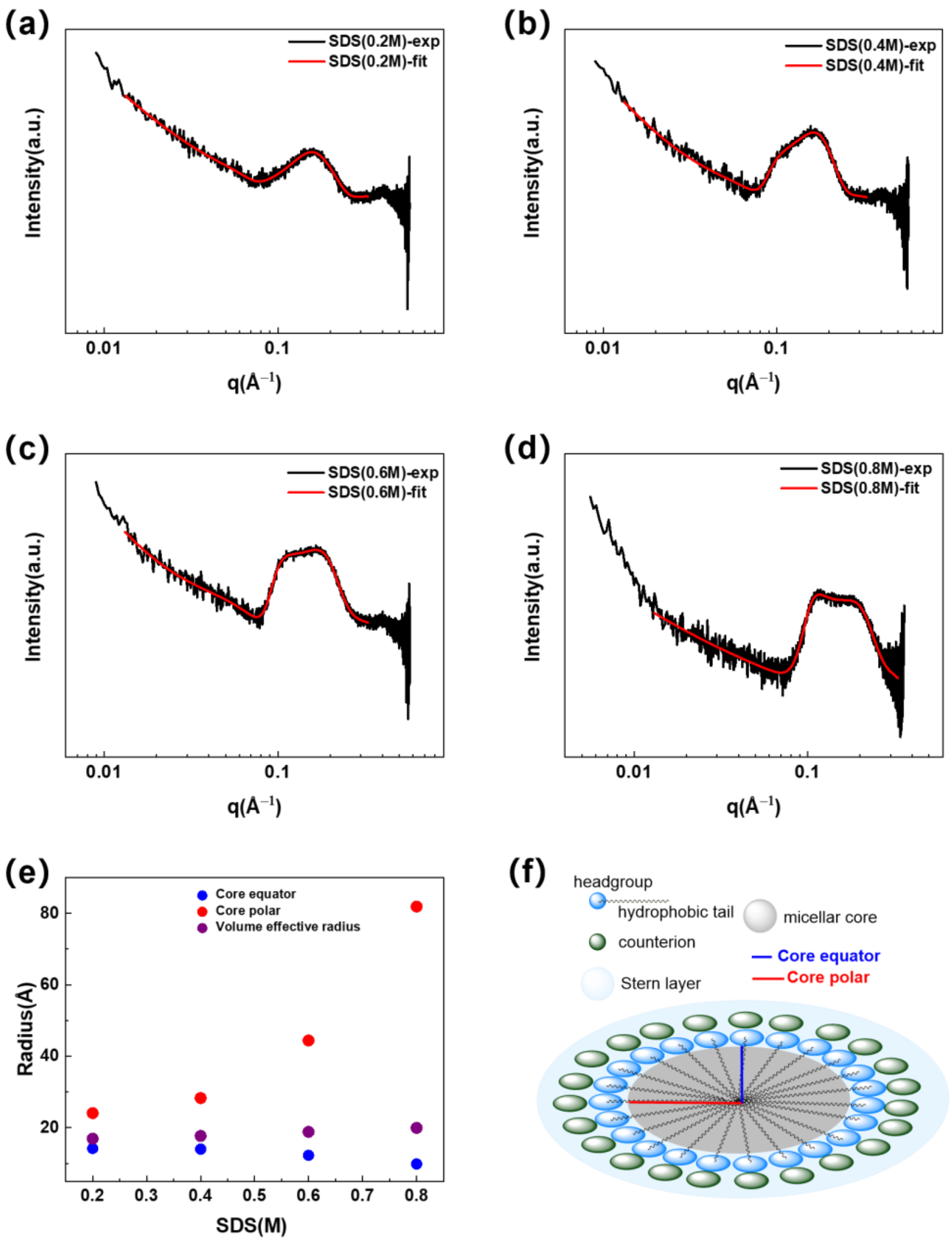

Figure S5: SAXS analysis of SDS micellar solutions (0.2–0.8 M) containing 50 mM Sub-1, 2.5 mM PC, and 100 mM DIPEA. **(a-d)** Experimental SAXS data (black line) and corresponding model fits (red line) for each SDS concentration. **(g)** Plot showing key micellar dimensions (core equator, core polar) and the volume effective interaction radius as a function of SDS concentration. **(h)** Schematic illustration of the core-shell ellipsoid model used for fitting.

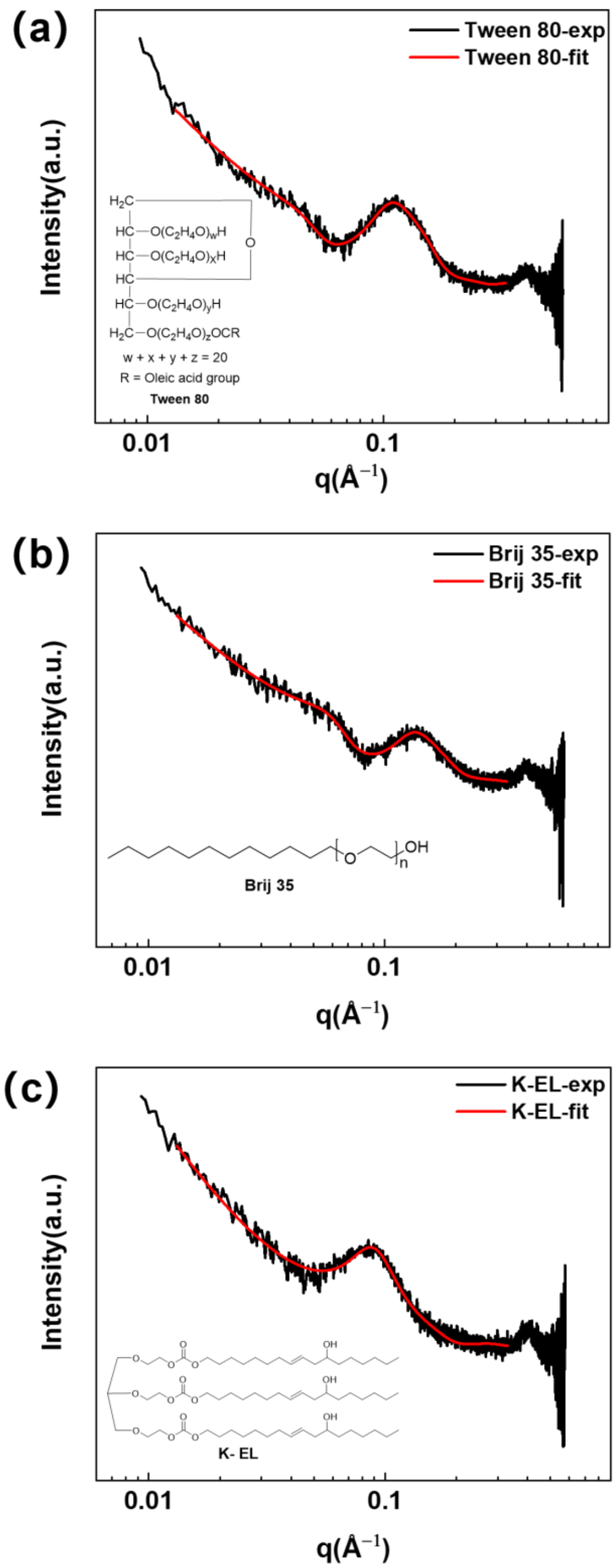

Figure S6: SAXS analysis of micellar solutions formed by different non-ionic surfactants (10 wt% in H_2O), each containing 50 mM Sub-1, 2.5 mM PC, and 100 mM DIPEA. Each panel displays the experimental SAXS data (black line), the corresponding model fit (red line): **(a)** Tween 80, **(b)** Brij 35, and **(c)** K-EL. The insets provide a structures of Tween 80, Brij 35 and K-EL.

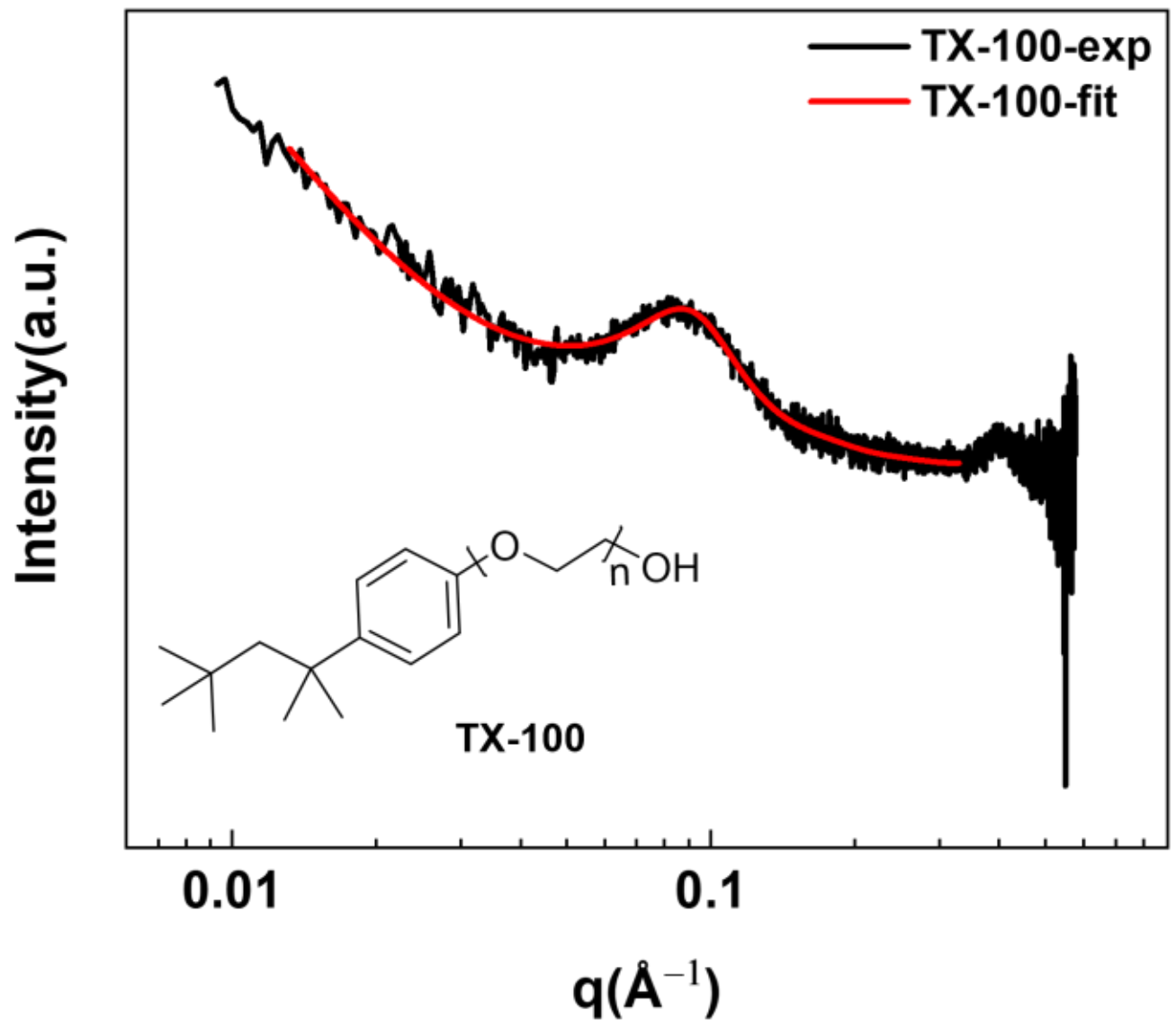

Figure S7: SAXS analysis of a TX-100 micellar solution (10 wt% in H_2O) containing 50 mM Sub-1, 2.5 mM PC, and 100 mM DIPEA. The plot displays the experimental SAXS data (black line) and the corresponding model fit (red line). The inset provides a structure of TX-100.

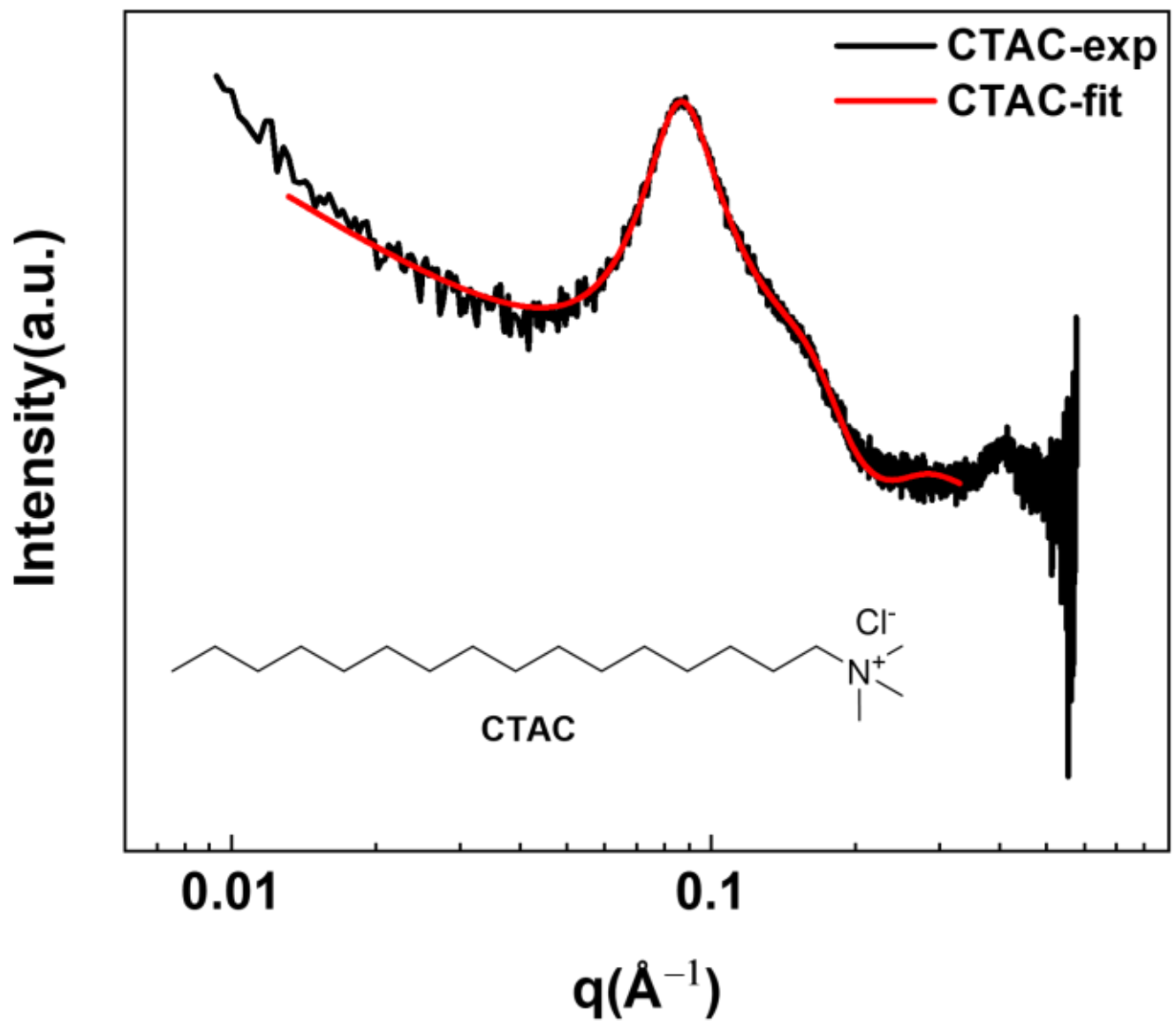

Figure S8: SAXS analysis of a CTAC micellar solution (0.4 M in H_2O) containing 50 mM Sub-1, 2.5 mM PC, and 100 mM DIPEA. The plot shows the experimental SAXS data (black line) along with the corresponding model fit (red line). The inset provides a structure of CTAC.

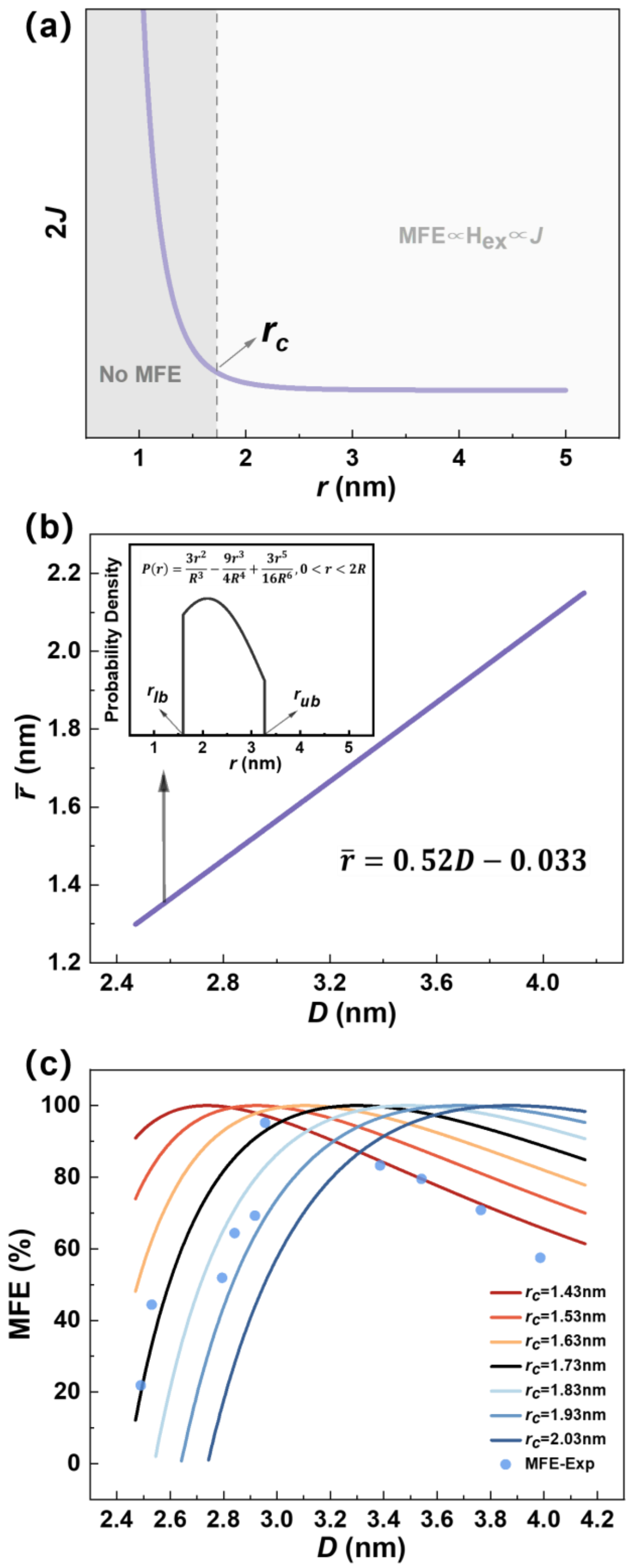

Figure S9: Theoretical simulation of the MFE based on RIP dynamics within a micelle. **(a)** The dependence of the spin-exchange interaction energy ($2J$) on the inter-radical distance (r). **(b)** The calculated average separation of the radical ion pair, $\bar{r}$, as a function of the micelle diameter, D. **(c)** The simulated MFE as a function of micelle diameter for several different values of the exchange interaction cut-off distance, r_c.

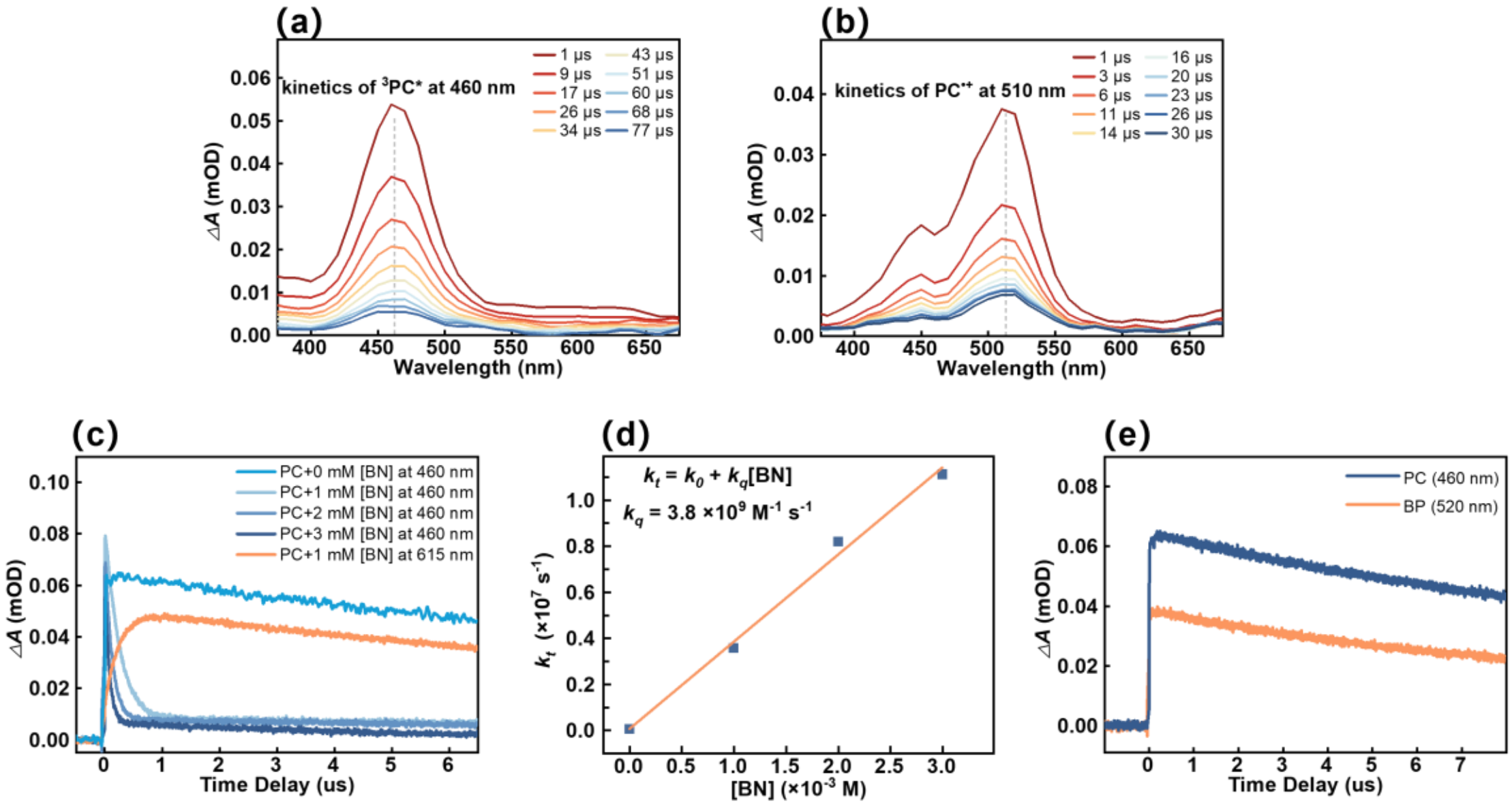

Figure S10: (**a**) Transient absorption spectra (TAS) for PC (1 mM) under N_2-saturated conditions; (**b**)Transient absorption spectra (TAS) for PC (1 mM) under air conditions; (**c**)Transient absorption kinetics at 460 nm at different concentrations of BN under N_2-saturated conditions; (**d**) Stern-Volmer plot obtained from the fitting of the kinetics at 460 nm with different concentrations of BN; (**e**) A comparison of the kinetics of ^{3}PC* at 460 nm and ^{3}BP* at 520 nm under N_2-saturated conditions. Experimental conditions: 355 nm laser excitation, solvent: MeCN.

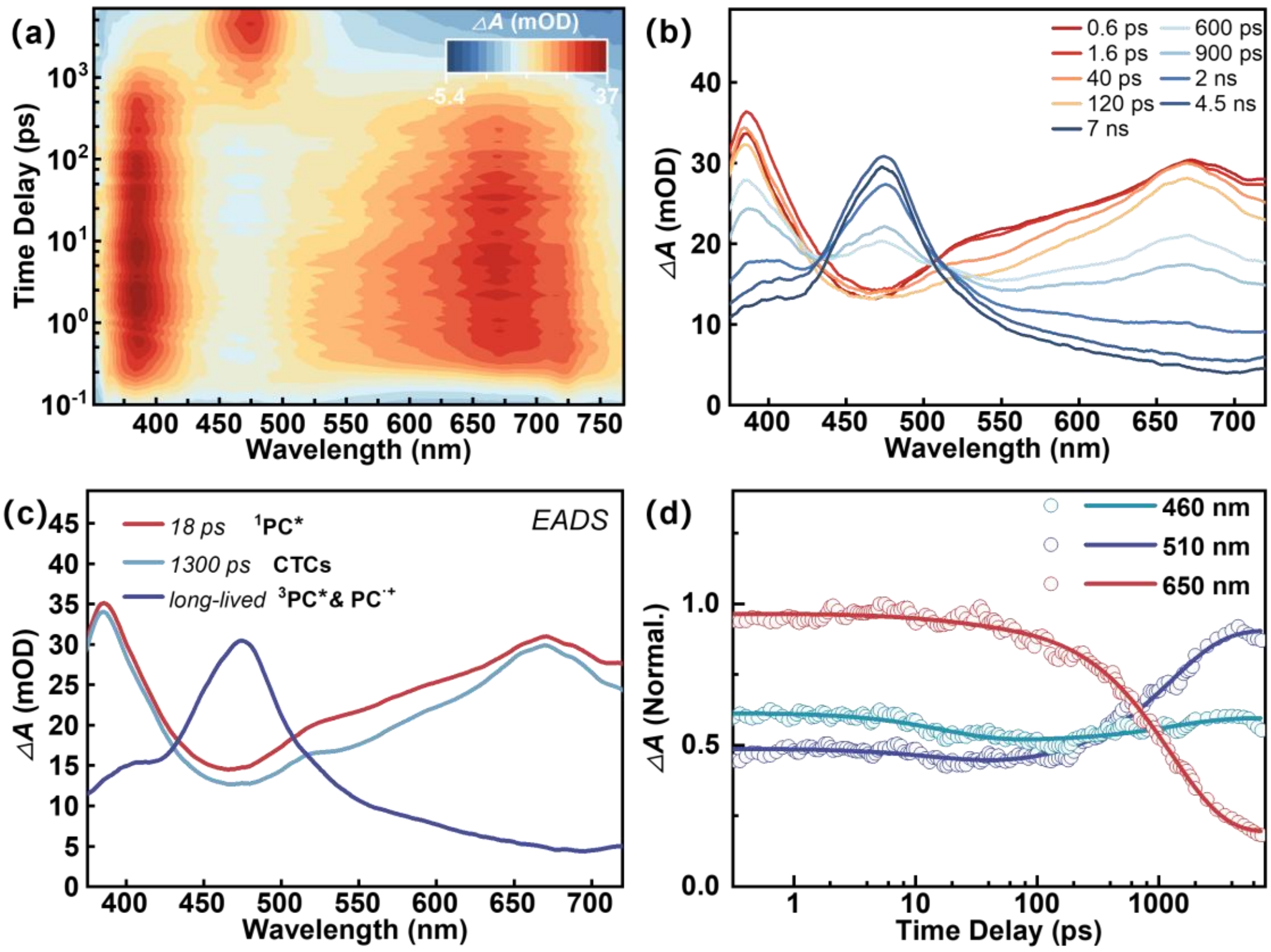

Figure S11: Time-Resolved Spectroscopy Experiment of PC (5 mM) + Sub-1 (1 M) in DMSO with 355 nm laser excitation. (**a**) Transient absorption two-dimension (2D) contour map; (**b**)TAS at typical delay times; (**c**) Evolution-associated differential spectra (EADS) obtained from the global analysis of the transient spectra; (**d**) Time traces at typical wavelengths (dots), together with the fits (lines) from the global analysis.

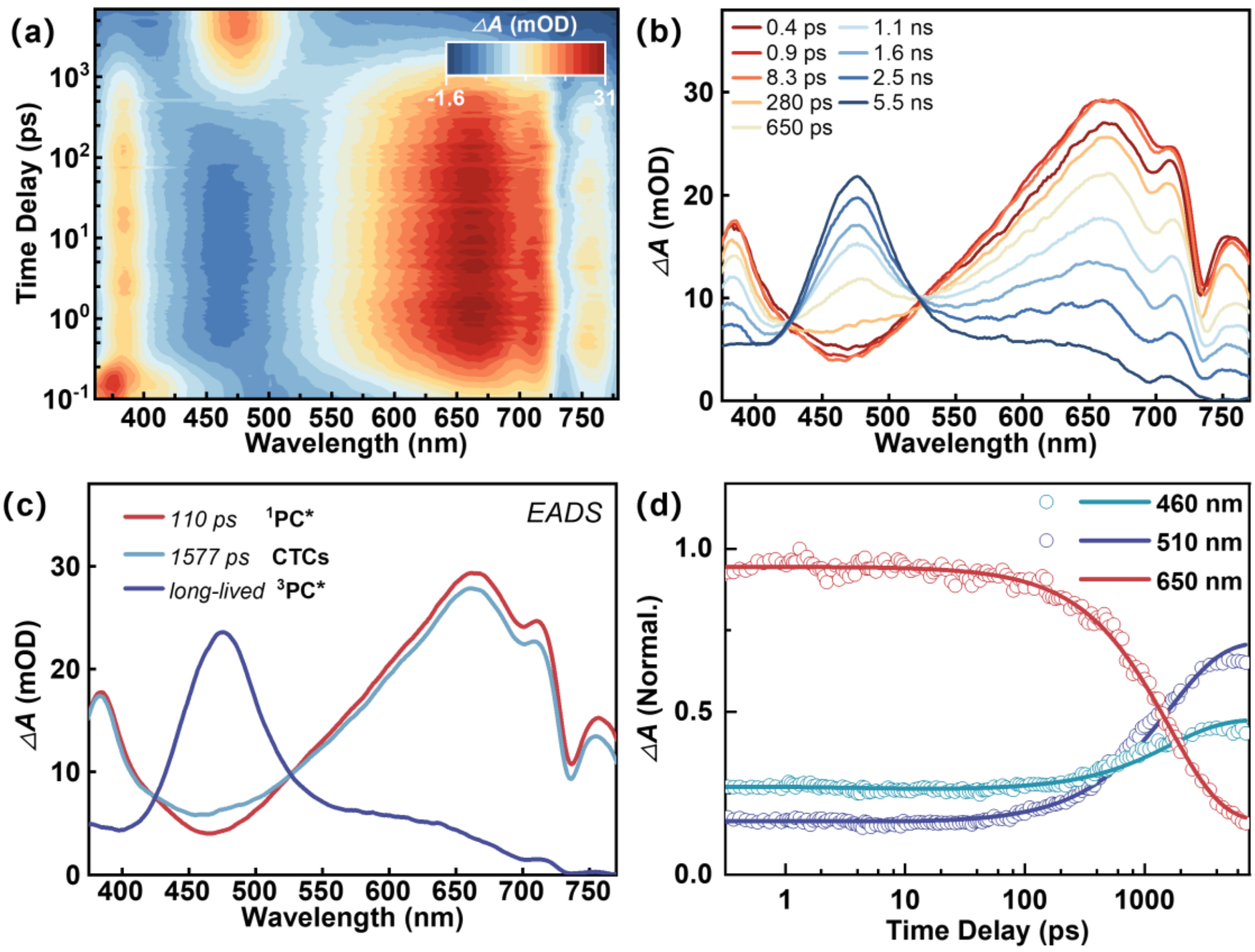

Figure S12: Time-Resolved Spectroscopy Experiment of PC (5 mM) + Sub-1 (1 M) + DIPEA (50 mM) in toluene with 355 nm laser excitation. (**a**) Transient absorption two-dimension (2D) contour map; (**b**)TAS at typical delay times; (**c**) Species-Associated Differential Spectra (SADS) obtained from the global analysis of the transient spectra; (**d**) Time traces at typical wavelengths (dots), together with the fits (lines) from the global analysis.

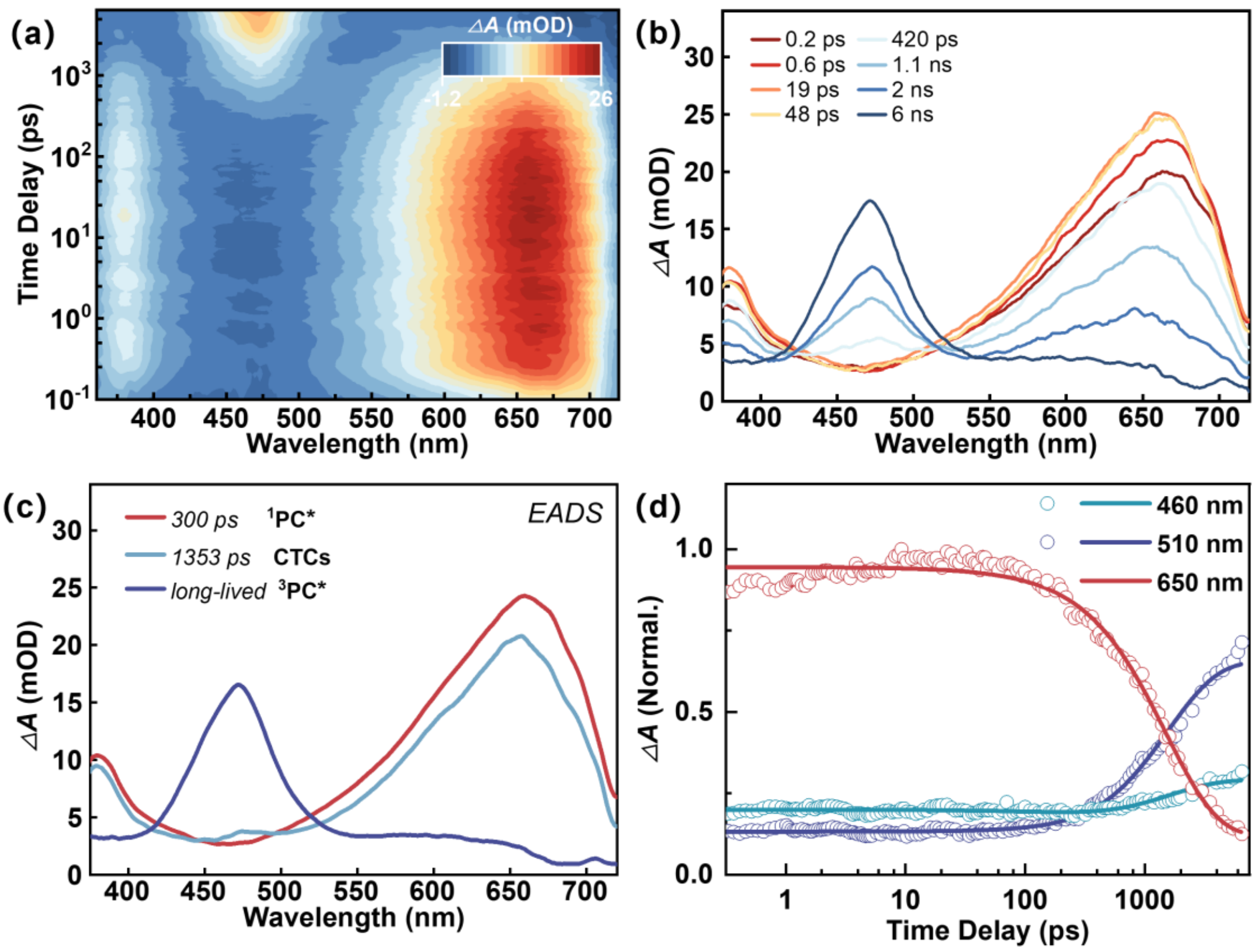

Figure S13: Time-Resolved Spectroscopy Experiment of PC (5 mM) + Sub-1 (1 M) + DIPEA (50 mM) in PEG-400 with 355 nm laser excitation. (**a**) Transient absorption two-dimension (2D) contour map; (**b**)TAS at typical delay times; (**c**) Evolution-associated differential spectra (EADS) obtained from the global analysis of the transient spectra; (**d**) Time traces at typical wavelengths (dots), together with the fits (lines) from the global analysis.

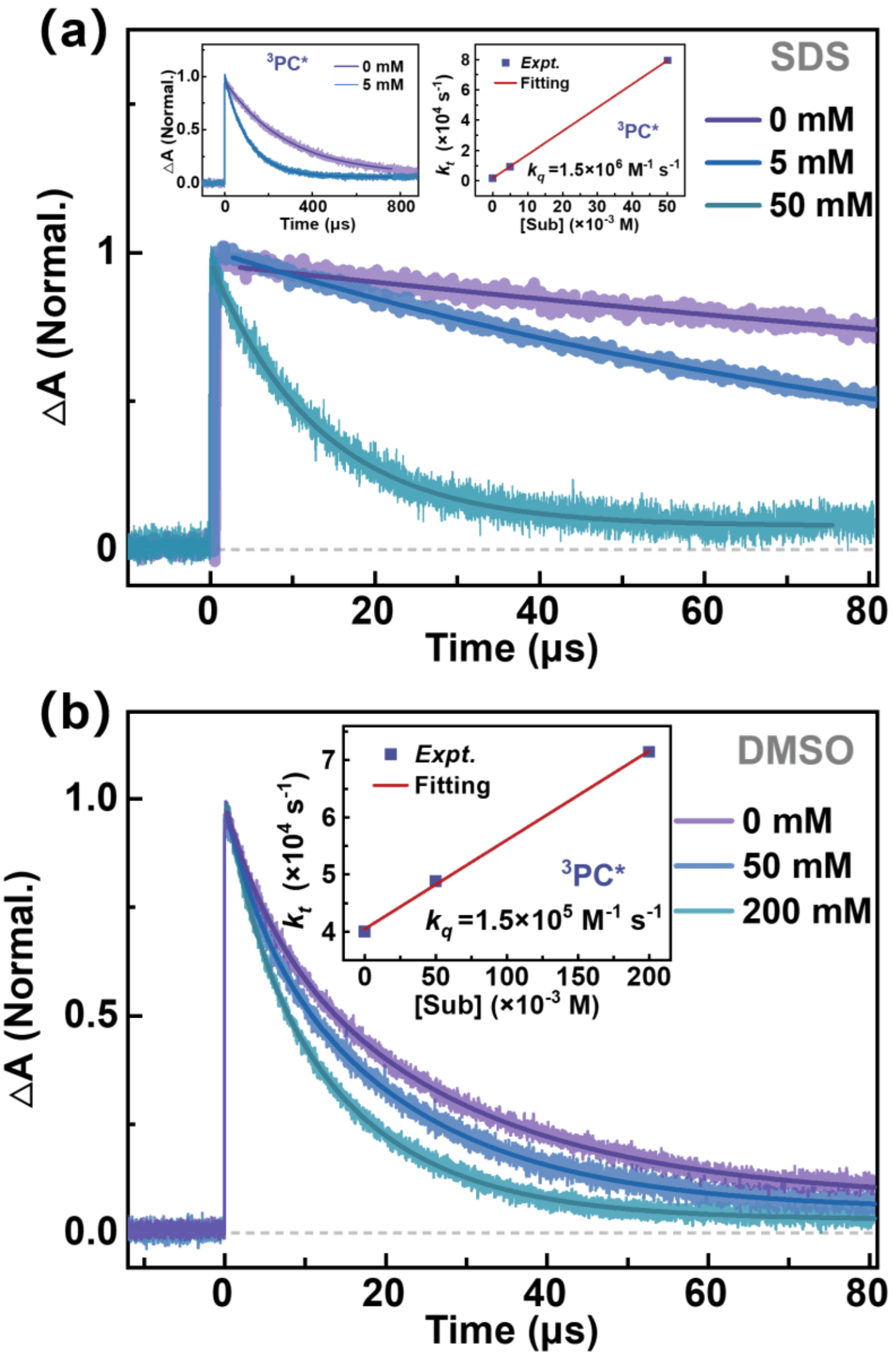

Figure S14: Decay kinetics of ^{3}PC* at 460 nm at different concentrations of Sub-1 in (**a**) SDS and (**b**) DSMO. Insert is the corresponding Stern-Volmer plot.

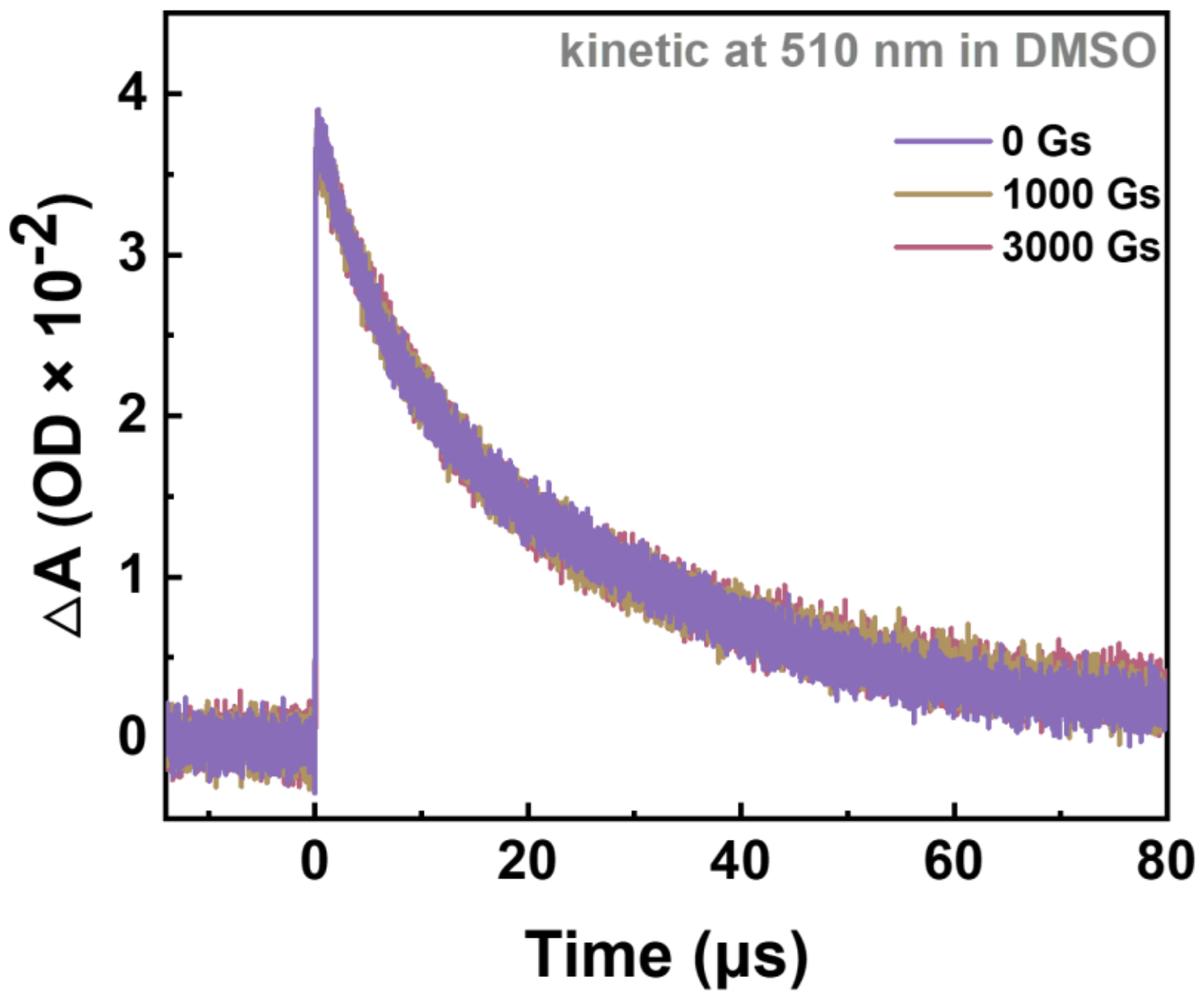

Figure S15: Photophysical and photochemical decay kinetics of $PC^{\bullet+}$ at 0 Gs and 3000 Gs in the presence of PC (5 mM) + Sub-1 (1 M) + DIPEA (50 mM) in DMSO with 355 nm laser excitation.

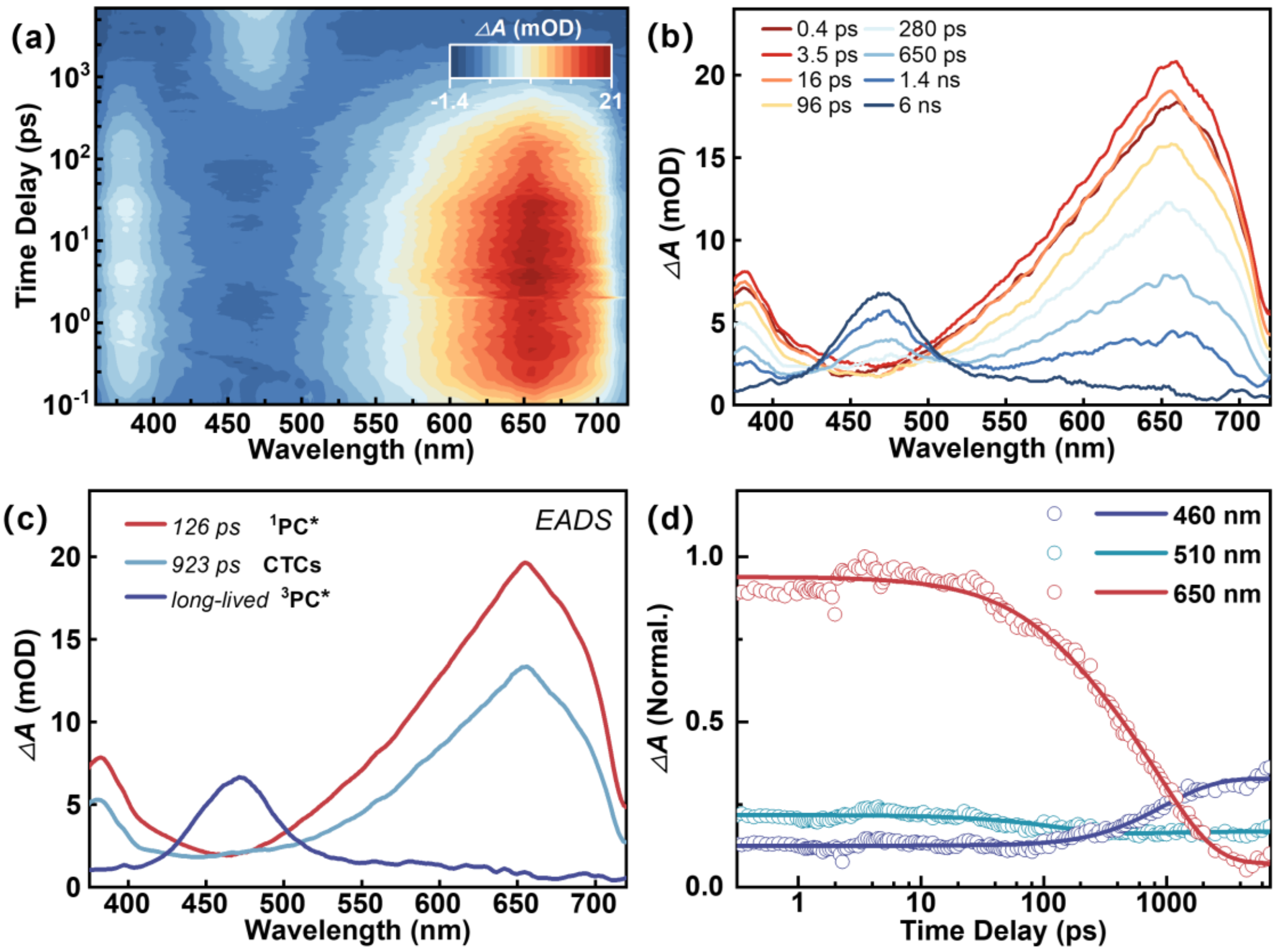

Figure S16: Time-Resolved Spectroscopy Experiment of PC (5 mM) + Sub-2 (50 mM) + DIPEA (50 mM) in SDS with 355 nm laser excitation. (**a**) Transient absorption two-dimension (2D) contour map; (**b**)TAS at typical delay times; (**c**) Evolution-associated differential spectra (EADS) obtained from the global analysis of the transient spectra; (**d**) Time traces at typical wavelengths (dots), together with the fits (lines) from the global analysis.

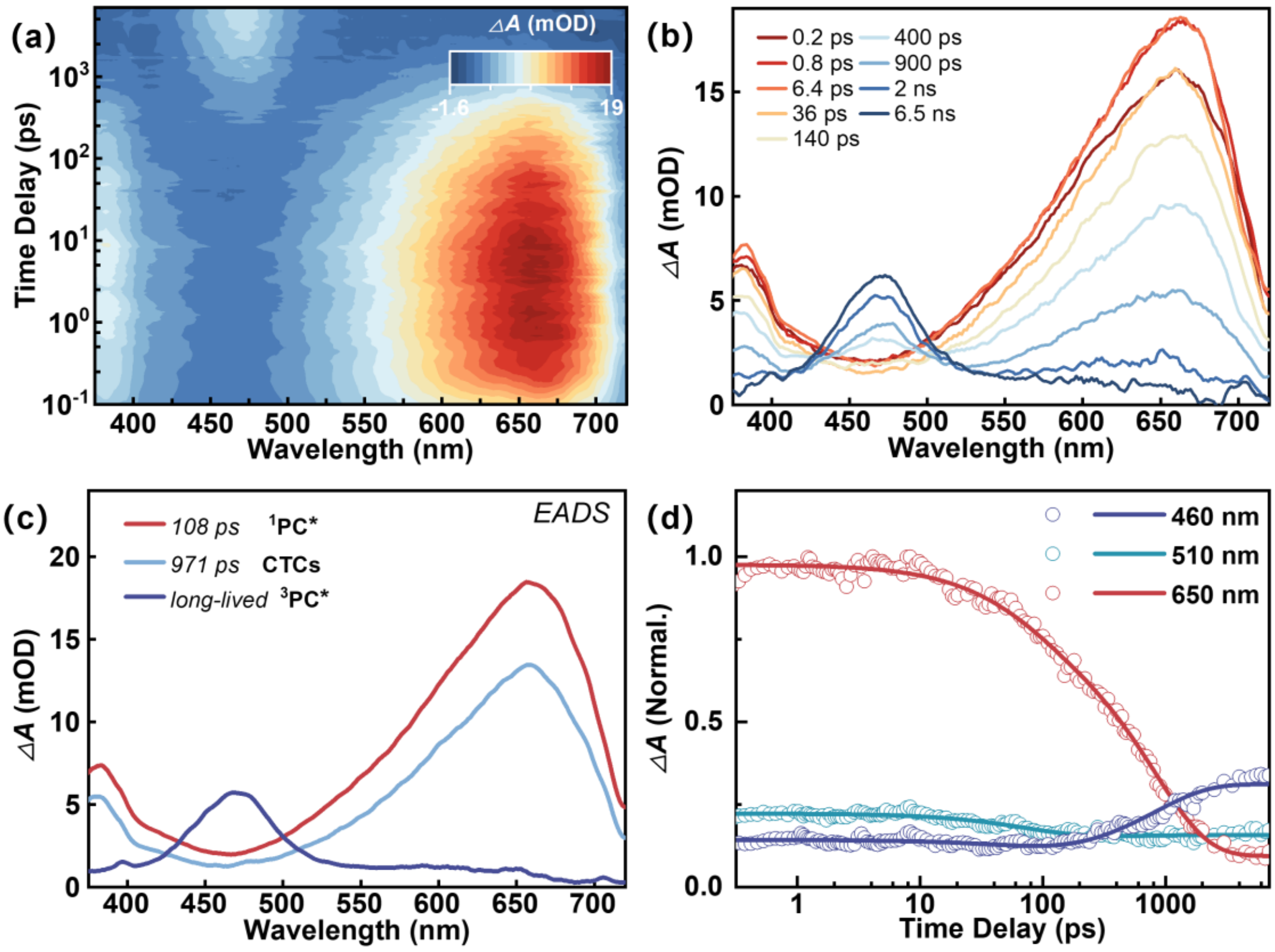

Figure S17: Time-Resolved Spectroscopy Experiment of PC (5 mM) + Sub-5 (50 mM) + DIPEA (50 mM) in SDS with 355 nm laser excitation. (**a**) Transient absorption two-dimension (2D) contour map; (**b**)TAS at typical delay times; (**c**) Evolution-associated differential spectra (EADS) obtained from the global analysis of the transient spectra; (**d**) Time traces at typical wavelengths (dots), together with the fits (lines) from the global analysis.

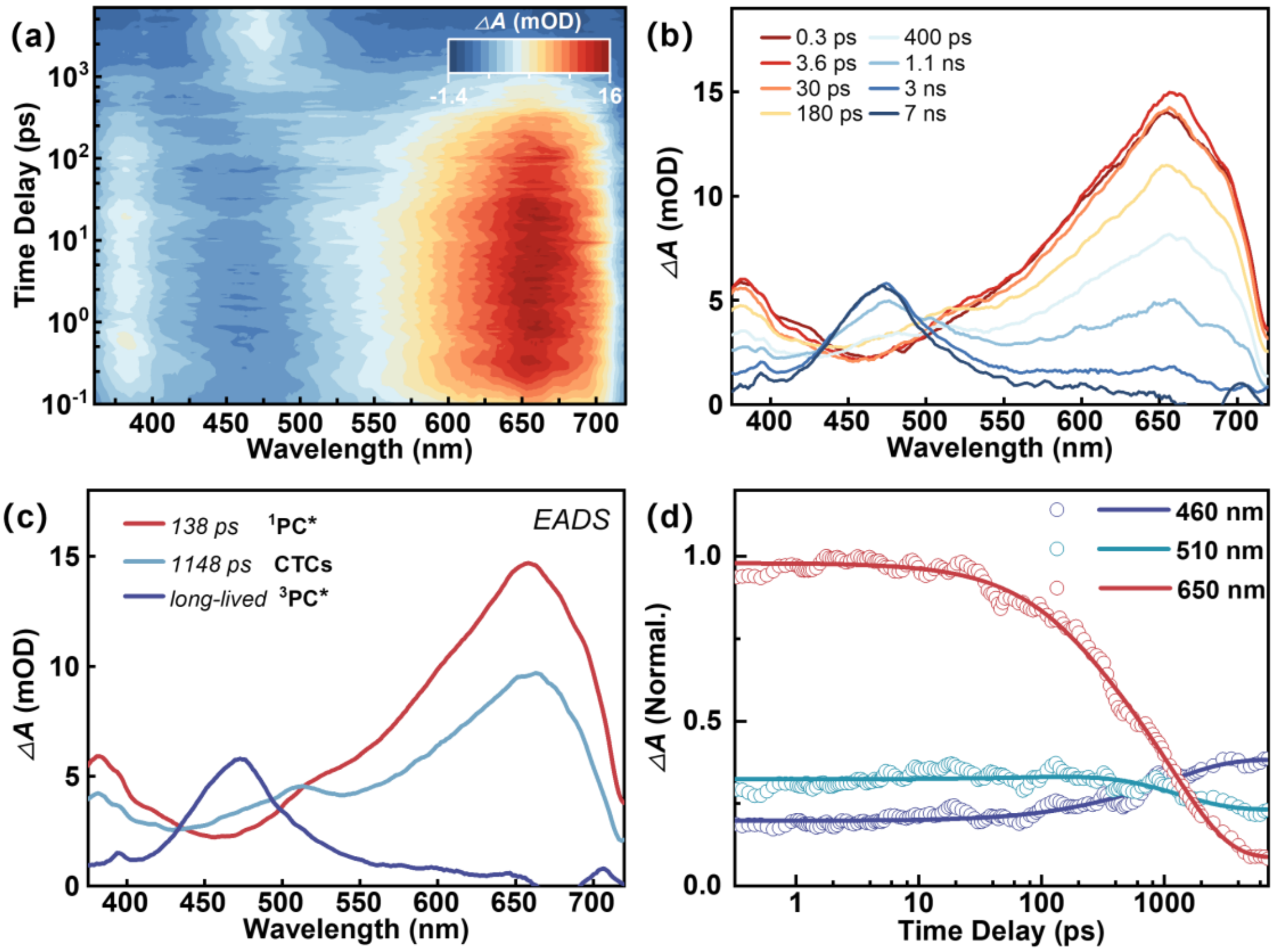

Figure S18: Time-Resolved Spectroscopy Experiment of PC (5 mM) + Sub-6 (50 mM) + DIPEA (50 mM) in SDS with 355 nm laser excitation. (**a**) Transient absorption two-dimension (2D) contour map; (**b**)TAS at typical delay times; (**c**) Evolution-associated differential spectra (EADS) obtained from the global analysis of the transient spectra; (**d**) Time traces at typical wavelengths (dots), together with the fits (lines) from the global analysis.

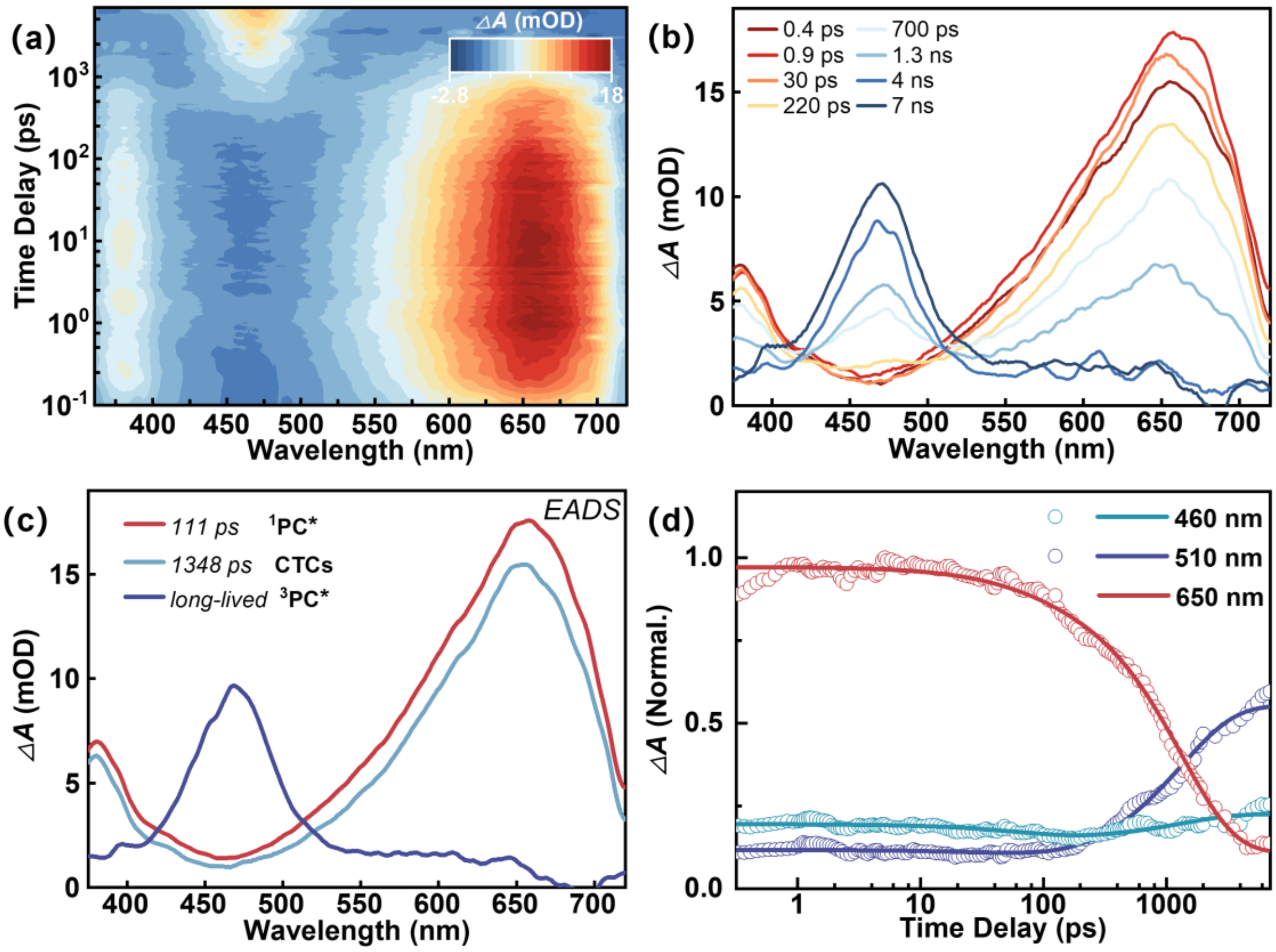

Figure S19: Time-Resolved Spectroscopy Experiment of PC (5 mM) + Sub-7 (50 mM) + DIPEA (50 mM) in SDS with 355 nm laser excitation. (**a**) Transient absorption two-dimension (2D) contour map; (**b**)TAS at typical delay times; (**c**) Evolution-associated differential spectra (EADS) obtained from the global analysis of the transient spectra; (**d**) Time traces at typical wavelengths (dots), together with the fits (lines) from the global analysis.

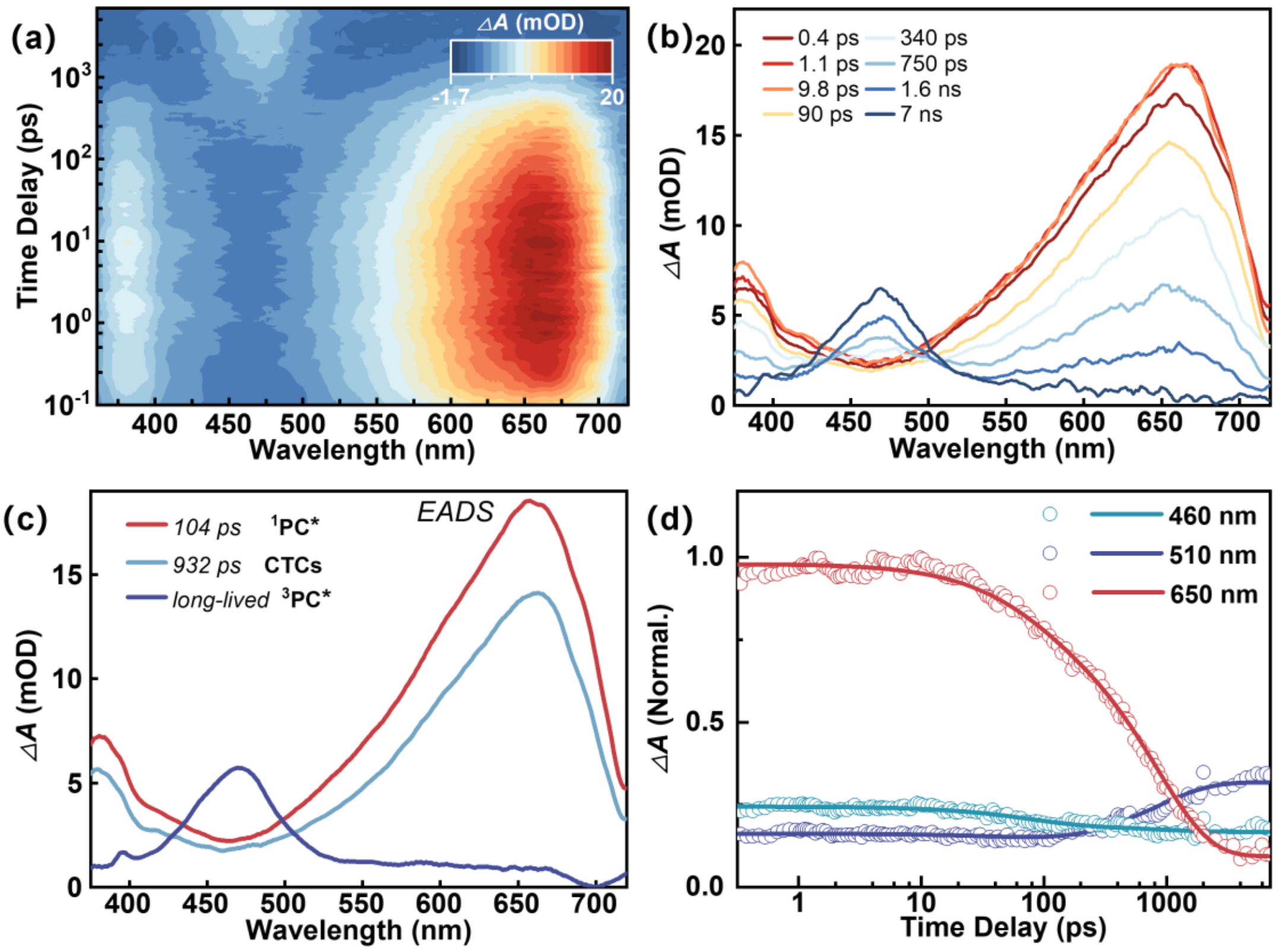

Figure S20: Time-Resolved Spectroscopy Experiment of PC (5 mM) + Sub-8 (50 mM) + DIPEA (50 mM) in SDS with 355 nm laser excitation. (**a**) Transient absorption two-dimension (2D) contour map; (**b**)TAS at typical delay times; (**c**) Evolution-associated differential spectra (EADS) obtained from the global analysis of the transient spectra; (**d**) Time traces at typical wavelengths (dots), together with the fits (lines) from the global analysis.

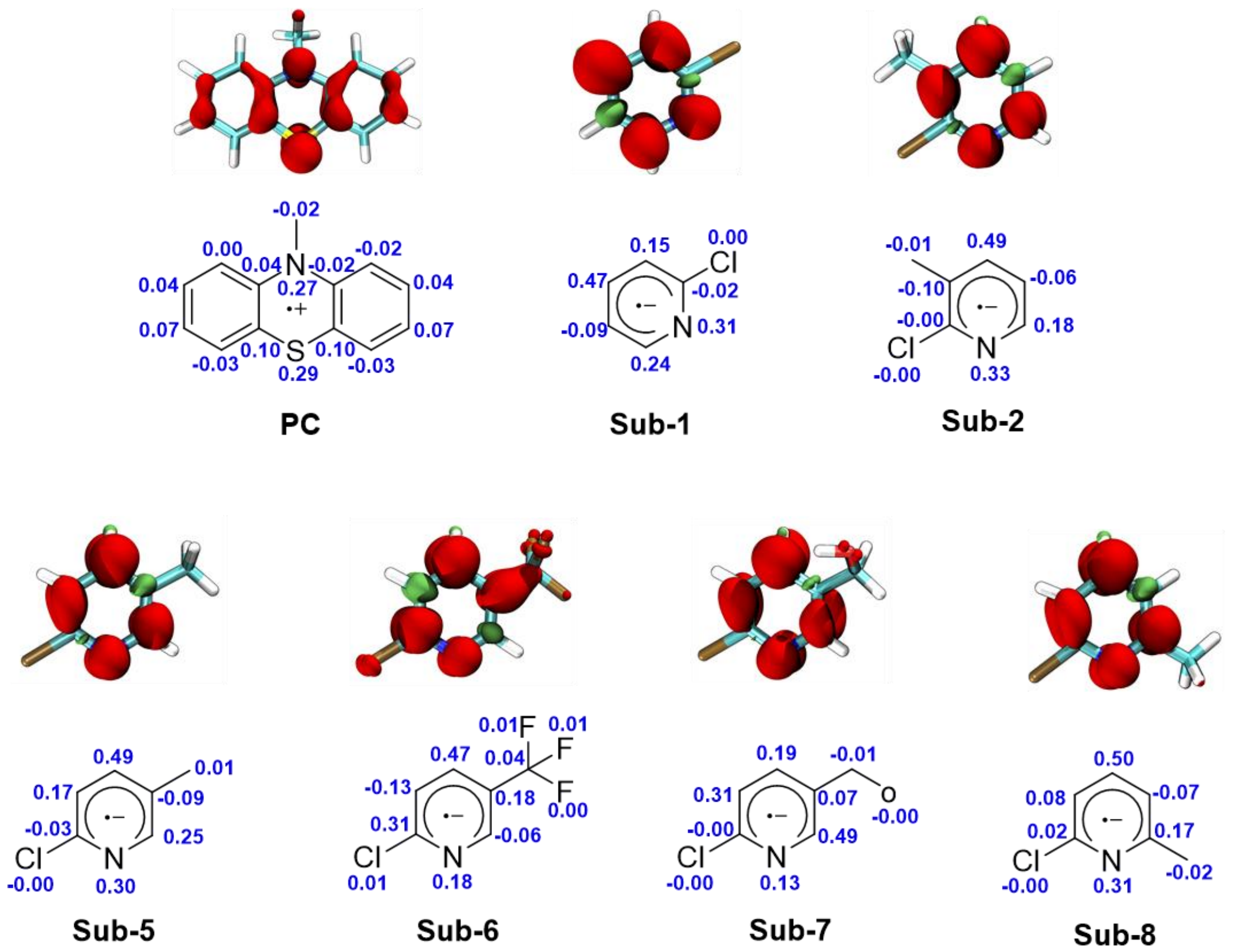

Figure S21. Calculated spin density distribution (Isovalue = 0.003) and Mulliken spin density (msd) values for PC and substrates at UB3LYP/def2-TZVP level.

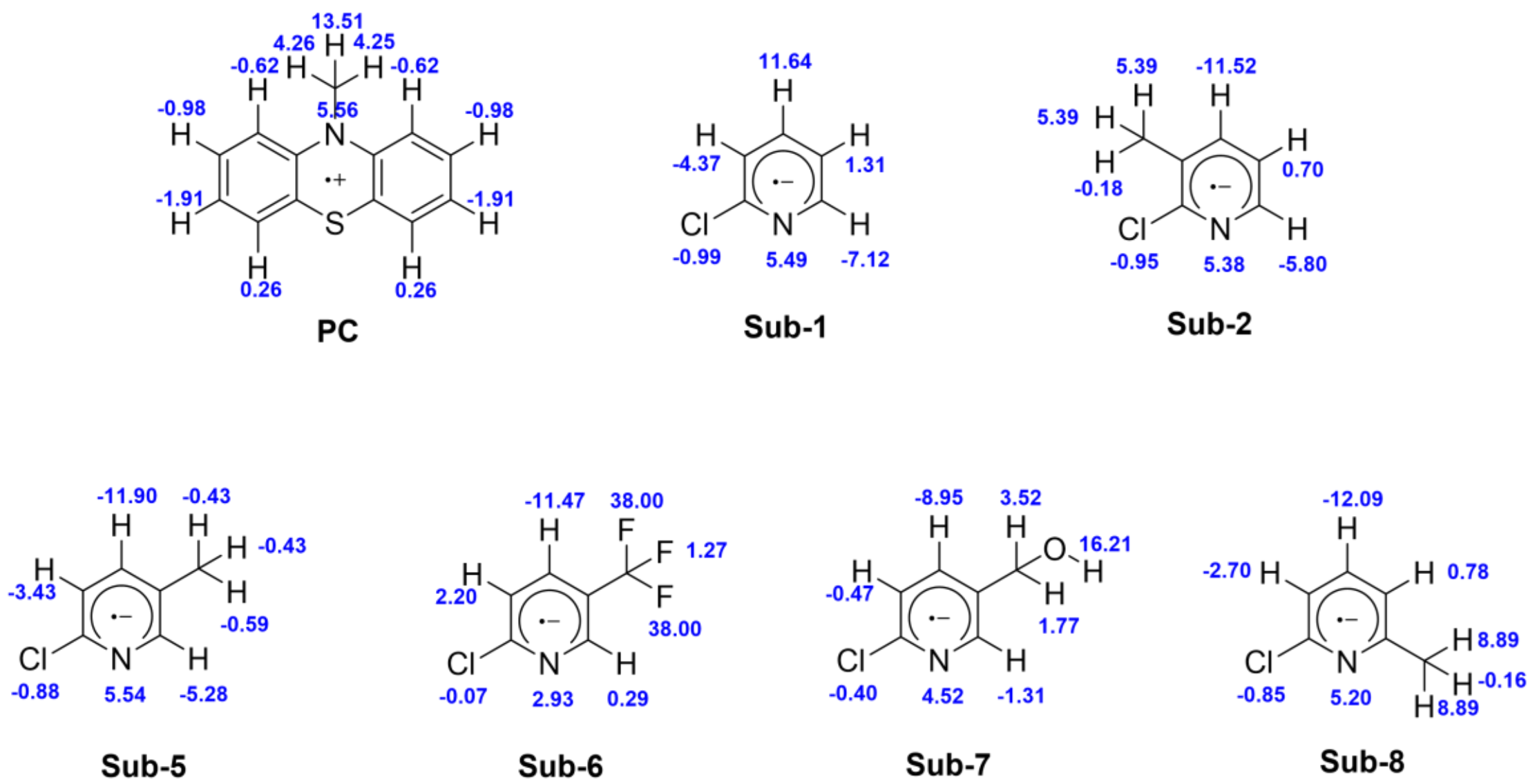

Figure S22. Calculated hyperfine coupling constants (HFCCs) (unit: Gauss) at UB3LYP/pcH-2 level.

Table S1 Macroscopic viscosity and corresponding MFE of various solvent and micellar systems.

Solvent	η(cP)[a]	MFE(%)[b]
DMSO	2.1	1.18
PEG-400	30.3	19.27
ILs/DMSO(V:V = 1:19)	2.4	1.86
PEG-400/H_2O(V:V = 3:8)	2.4	1.26
SDS(0.4 M)	2.4	79.44
K-EL(10 wt%)	2.2	5.16
TX-100(10 wt%)	3.5	25.53
Tween 80(10 wt%)	2.1	29.78
CTAC(0.4 M)	2.9	7.84
Brij 35(10 wt%)	3.1	9.03

[a] The average value of three independent measurements at room temperature (25 °C); [b] The magnetic field effect (MFE) values measured under an external magnetic field of 3000 Gs.

Note: The macroscopic viscosity of all systems was measured to evaluate its potential contribution to the observed MFEs. As shown, while most micellar systems and mixed solvents exhibit similar macroscopic viscosities (in the range of 2.1–3.5 cP, excluding pure PEG-400), their corresponding MFEs vary significantly. Notably, the SDS (0.4 M) system delivers a remarkably high MFE of 79.44% at a viscosity of 2.4 cP, which is identical to the viscosity of the PEG-400/H_2O mixture (2.4 cP, MFE = 1.26%). This stark contrast unambiguously rules out macroscopic viscosity as the primary contributor to the prominent MFEs observed in micellar confinement.

Table S2 The fitting parameters of SDS(0.01-0.1 M).

**SDS(M)**	0.01	0.02	0.04	0.06	0.08	0.1
**Radius-effective (Å)**	13.221	13.515	14.361	14.505	15.399	15.467
**Radius (Å)**	12.453	12.659	13.975	14.21	14.592	14.78
**Charge**	11.081	199.83	10.576	7.644	9.8173	9.513
**Volume fraction**	0.24985	0.19497	0.24232	0.15523	0.21432	0.20743
**Power**	1.9485	1.6865	1.7981	2.0153	1.9525	2.0114

Table S3 The fitting parameters of SDS(0.2-0.8 M).

SDS(M)	0.2	0.4	0.6	0.8
**Radius-core-equator(Å)**	14.202	14.01	12.256	9.8374
**Radius-x-core-polar**	1.6959	2.02	3.6225	8.3236
**Thickness-shell-equator(Å)**	12.164	15.572	17.429	26.116
**Thickness-x-shell-polar**	0.70747	0.5521	0.54773	0.24475
**Radius-effective(Å)**	14.071	17.696	18.417	23.548
**Volume fraction**	0.01099	0.0447	0.06489	0.18409
**Charge**	16.962	19.368	56.662	26.054
**Radius-Core-polar(Å)**	24.08517	28.3002	44.39736	81.88258
**Thickness-shell-polar(Å)**	8.60567	8.5973	9.54639	6.39189
**Power**	1.5852	1.8764	2.1066	1.1901
**Volume effective radius(Å)**	16.93621	17.71014	18.82283	19.93658

Table S4 The fitting parameters of Tween 80, Brij 35 and K-EL.

Micelles	Tween 80	Brij 35	K-EL
**Radius-core(Å)**	16.589	14.276	20.831
**Shell-thickness(Å)**	30.007	24.548	0.000000039
**Radius-effective(Å)**	57.484	46.895	33.718
**Volulme fraction**	0.24022	0.25003	0.27739
**Power**	1.6466	1.5224	1.7845

Table S5 The fitting parameters of TX-100.

Micelles	TX-100
**Radius-core(Å)**	14.361
**Shell-thickness(Å)**	17.216
**Radius-effective(Å)**	32.617
**Volulme fraction**	0.25003
**Power**	1.5224
**Radius**	-

Table S6 The fitting parameters of CTAC.

Micelles	CTAC
**Radius(Å)**	19.466
**Radius-effective(Å)**	34.126
**Power**	1.0814
**Charge**	21.994
**Volulme fraction**	0.32679

Table S7 Reaction conditions of photocatalytic measurements for different substrates.

Sub	PC	Base	Solvent	Time	Optical Power	Yield (0 Gs)	Yield (3000 Gs)
Sub-1(0.05 M)	PC(2.60 mM)	DIPEA(0.1 M)	Tol	15 min	145 mW	0.00	0.00
Sub-1(0.05 M)	PC(3.00 mM)	DIPEA(0.1 M)	THF	15 min	145 mW	0.00	0.00
Sub-1(0.05 M)	PC(2.60 mM)	DIPEA(0.1 M)	DCE	15 min	145 mW	0.00	0.00
Sub-1(0.05 M)	PC(3.82 mM)	DIPEA(0.1 M)	IPA	15 min	145 mW	0.00	0.00
Sub-1(0.05 M)	PC(3.16 mM)	DIPEA(0.1 M)	ACN	15 min	145 mW	0.00	0.00
Sub-1(0.05 M)	PC(1.88 mM)	DIPEA(0.1 M)	DMSO	15 min	145 mW	13.56	13.72
Sub-1(0.05 M)	PC(2.65 mM)	DIPEA(0.1 M)	PEG-400	15 min	145 mW	9.70	11.57
Sub-1(0.05 M)	PC(2.79 mM)	DIPEA(0.1 M)	K-EL(10 wt%)	15 min	145 mW	2.33	2.45
Sub-1(0.05 M)	PC(2.44 mM)	DIPEA(0.1 M)	TX-100(10 wt%)	7.5 min	145 mW	8.67	10.88
Sub-1(0.05 M)	PC(2.28 mM)	DIPEA(0.1 M)	Tween 80(10 wt%)	15 min	145 mW	3.48	4.52
Sub-1(0.05 M)	PC(2.55 mM)	DIPEA(0.1 M)	Brij 35(10 wt%)	3 min	145 mW	6.82	7.43
Sub-1(0.05 M)	PC(2.50 mM)	DIPEA(0.1 M)	SDS(0.4M)	2.5 min	145 mW	6.18	11.08
Sub-1(0.05 M)	PC(2.10 mM)	DIPEA(0.1 M)	CTAC(0.4M)	3 min	145 mW	7.65	8.25
Sub-1(0.05 M)	PC(2.50 mM)	DIPEA(0.1 M)	SDS(0.8M)	3 min	145 mW	7.93	12.49
Sub-1(0.05 M)	PC(2.50 mM)	DIPEA(0.1 M)	SDS(0.6M)	2 min	145 mW	5.32	9.08
Sub-1(0.05 M)	PC(2.50 mM)	DIPEA(0.1 M)	SDS(0.2M)	2.5 min	145 mW	5.81	10.45
Sub-1(0.05 M)	PC(2.50 mM)	DIPEA(0.1 M)	SDS(0.1M)	3 min	145 mW	5.69	11.10
Sub-1(0.05 M)	PC(2.50 mM)	DIPEA(0.1 M)	SDS(0.08M)	5 min	145 mW	6.10	10.32
Sub-1(0.05 M)	PC(2.50 mM)	DIPEA(0.1 M)	SDS(0.06M)	5 min	145 mW	6.82	11.20
Sub-1(0.05 M)	PC(2.50 mM)	DIPEA(0.1 M)	SDS(0.04M)	5 min	145 mW	4.76	7.23
Sub-1(0.025 M)	PC(1.25 mM)	DIPEA(0.05 M)	SDS(0.02M)	7 min	145 mW	6.39	9.23
Sub-1(0.025 M)	PC(1.25 mM)	DIPEA(0.05 M)	SDS(0.01M)	10 min	145 mW	4.80	5.85
Sub-1(0.05 M)	PC(2.50 mM)	DIPEA(0.1 M)	SDS(0.4M)	2.5 min	145 mW	6.18	11.08
Sub-2(0.05 M)	PC(2.50 mM)	DIPEA(0.1 M)	SDS(0.4M)	6 min	145 mW	7.44	15.16
Sub-3(0.05 M)	PC(2.50 mM)	DIPEA(0.1 M)	SDS(0.4M)	7 min	145 mW	6.98	11.70
Sub-4(0.05 M)	PC(2.50 mM)	DIPEA(0.1 M)	SDS(0.4M)	10 min	145 mW	4.74	8.16
Sub-5(0.05 M)	PC(2.50 mM)	DIPEA(0.1 M)	SDS(0.4M)	5 min	145 mW	5.39	11.79
Sub-6(0.05 M)	PC(2.50 mM)	DIPEA(0.1 M)	SDS(0.4M)	7 min	145 mW	7.86	14.75
Sub-7(0.05 M)	PC(2.50 mM)	DIPEA(0.1 M)	SDS(0.4M)	2 min	145 mW	9.42	13.25
Sub-8(0.05 M)	PC(2.50 mM)	DIPEA(0.1 M)	SDS(0.4M)	7 min	145 mW	5.65	10.69
Sub-9(0.05 M)	PC(2.50 mM)	DIPEA(0.1 M)	SDS(0.4M)	8 min	145 mW	14.67	18.03
Sub-10(0.05 M)	PC(2.50 mM)	DIPEA(0.1 M)	SDS(0.4M)	4 min	145 mW	6.69	14.06
Sub-11(0.05 M)	PC(2.50 mM)	DIPEA(0.1 M)	SDS(0.4M)	1.5 min	145 mW	7.55	9.09
Sub-12(0.05 M)	PC(2.50 mM)	DIPEA(0.1 M)	SDS(0.4M)	10 min	145 mW	10.07	13.44

The catalyst concentrations are different in different reaction solvents to ensure that the absorbance at 375 nm is the same, which meets the condition for comparing the initial reaction rates.

Table S8 Parameters for quantitative analysis of magnetic field effects for different substrates

Sub	Sub-1	Sub-2	Sub-5	Sub-6	Sub-7	Sub-8
$B_{Sub^{\cdot-}}$ [a]	14.80	15.17	14.17	47.82	17.65	17.05
$B_{PC^{\cdot+}}$ [a]	15.28	15.28	15.28	15.28	15.28	15.28
$B_{1/2}^{\mathrm{Theo}}$(Gs) [b]	30.09	30.45	29.49	79.89	33.10	32.43
$k_{RIP-ISC}(0)$ [c]	2.65E+08	2.68E+08	2.60E+08	7.03E+08	2.91E+08	2.85E+08
$k_{RIP-ISC}(B_{sat})$ [c]	8.83E+07	8.93E+07	8.65E+07	2.34E+08	9.71E+07	9.51E+07
$\tau_{1_{PC*}}$(ps) [d]	1500	1500	1500	1500	1500	1500
$\tau_{1_{PC*}}$(ps) [e]	65	126	108	138	111	104
τ_{CTC}/ps [f]	870	923	971	1148	1348	932
k_{QCTC}/s^{-1} [g]	1.15E+09	1.08E+09	1.03E+09	6.76E+09	7.42E+08	1.07E+09
f_{TCR} [h]	0.51	0.35	0.28	0.36	0.51	0.27
k_{TCR}/s^{-1} [h]	5.86E+08	3.79E+08	2.88E+08	2.43E+09	3.78E+08	2.90E+08
f_{BET} [i]	0.47	0.6474	0.719	0.625	0.42	0.727
k_{BET}/s^{-1} [i]	5.40E+08	7.01E+08	7.40E+08	4.22E+09	3.12E+08	7.80E+08
$f_{RIP-ISC}(0)$ [j]	0.920	0.990	0.996	0.874	0.849	0.989
$P^S(0)$ [k]	0.038	0.004	0.001	0.022	0.123	0.004
$P^T(0)$ [k]	0.077	0.006	0.002	0.066	0.136	0.004
$P(0)$ [k]	0.114	0.010	0.003	0.088	0.259	0.008
$f_{RIP-ISC}(B)$ [j]	0.793	0.969	0.988	0.698	0.652	0.967
$P^S(B)$ [k]	0.034	0.004	0.001	0.020	0.105	0.004
$P^T(B)$ [k]	0.177	0.016	0.005	0.145	0.266	0.012
$P(B)$ [k]	0.211	0.020	0.006	0.165	0.371	0.016
$\mathrm{MFE_{Yield}}(B_{sat})$/% [i]	80.52	110.76	122.61	89.21	41.82	94.74
f_{CE} [i]	0.02	0.0026	0.001	0.015	0.07	0.003
k_{CE}/s^{-1} [i]	2.30E+07	2.82E+06	1.03E+06	1.01E+08	5.19E+07	3.22E+06
$B_{1/2}^{k_{RIP-ISC}}$(Gs) [l]	40	90	70	100	30	70
$B_{1/2}^{Yield}$(Gs) [m]	90	300	250	250	30	200

[a] $B_i(i = PC^{\cdot+}, Sub^{\cdot-})$represents the effective hyperfine coupling strength of the radical cation and anion, respectively, calculated from the hyperfine coupling constants obtained via DFT calculations according to Equation (12).

[b] $B_{1/2}^{\mathrm{Theo}}$ is the theoretical half-saturation field, calculated using Equation (11).

[c] $k_{RIP-ISC}(0)$ is the ISC rate constant of radical ion pairs at zero field, estimated using Equation (10). $k_{RIP-ISC}(B)$ is the ISC rate constant at the saturation field, calculated using Equation (13).

[d] The lifetime of ^{1}PC* without Sub-1.

[e] The lifetime of ^{1}PC* with Sub-1.

[f] Lifetime of the CTC.

[g] Total decay rate constant of CTC, calculated as $k_{QCTC} = 1/\tau_{CTC}$.

[h] f_{TCR} is the branching ratio of the TCR pathway, determined directly from the global analysis of femtosecond transient absorption (fs-TA) data. k_{TCR} is the corresponding rate constant, calculated as $k_{TCR} = f_{TCR} * k_{QTCR}$.

[i] $\mathrm{MFE_{Yield}}(B_{sat})$ is the experimentally measured MFE on the yield at saturation. Combining Equations (1), (3), (4) and (9), an expression for $\mathrm{MFE_{Yield}}(B_{sat})$ is obtained where k_{CE} is the only unknown and solving this equation yields the value of k_{CE}. The branching ratio of cage escape, f_{CE}, was then calculated as $f_{CE} = k_{CE}/k_{QCTC}$. Consequently, the branching ratio of BET, f_{BET}, was determined using $f_{BET} = 1 - f_{TCR} - f_{CE}$, and its rate constant k_{BET} was calculated as $k_{BET} = f_{BET} * k_{QCTC}$.

[j] $f_{RIP-ISC}(0)$ and $f_{RIP-ISC}(B)$ are the branching ratios for the regeneration of ^{1}CTC from the RIP via ISC at zero field and saturation field, respectively, calculated using Equation (4).

[k] P^S, P^T, and P are the probabilities of effective free-radical formation via the singlet pathway, triplet pathway, and the total probability, respectively. (0) and (B) denote the values at zero field and saturation field, respectively, calculated using Equations (6), (8), and (9).

[l] Intrinsic half-saturation field. This value is obtained by fitting the curve of $k_{RIP-ISC}(B)$ versus the magnetic field B. The $k_{RIP-ISC}(B)$ values are back-calculated from the model using the experimental yield $P(B)$ at various intermediate magnetic fields. It represents the true magnetic sensitivity of the spin conversion process.

[m] Yield half-saturation field. This is a direct experimental observable, defined as the magnetic field strength at which the magnetic field effect on the total yield reaches half of its saturation value.

Table S9 Cartesian coordinates of optimized PC cation radical and substrate anion radicals

PC			
C	-3.734826	-0.674421	0.220117
C	-2.603123	-1.436701	0.048743
C	-1.346304	-0.826102	-0.057802
C	-1.224586	0.584299	-0.014523
C	-2.397919	1.336648	0.183215
C	-3.62264	0.719104	0.30021
C	1.224572	0.584268	-0.014497
C	1.346304	-0.826125	-0.057821
C	2.603123	-1.436716	0.048722
H	2.668495	-2.516009	0.003853
C	3.734821	-0.674427	0.220096
C	3.622628	0.719096	0.300229
C	2.3979	1.336628	0.183262
H	-4.701231	-1.150497	0.307131
H	-2.668483	-2.515997	0.003896
H	-2.345755	2.40883	0.278405
H	-4.504474	1.322553	0.465118
H	4.701232	-1.150498	0.307072
H	4.504457	1.322549	0.465149
H	2.345696	2.408806	0.278509
S	-0.000011	-1.880177	-0.283426
N	0.000004	1.218976	-0.171336
C	0.000053	2.657133	-0.473526
H	0.87734	2.89327	-1.065264
H	-0.876856	2.893201	-1.065857
H	-0.000281	3.257701	0.43761
Sub-1			
C	-2.242853	-0.053725	0.00001
C	-1.497744	-1.209466	0.000009
C	0.437227	-0.020141	-0.000042
C	-0.176762	1.204517	-0.000013
C	-1.624147	1.209573	0.000022
H	-1.974752	-2.183703	0.000043
H	0.405085	2.114797	0.000015
H	-2.187221	2.132538	-0.000069
Cl	2.2576	-0.006357	0.000014
H	-3.327262	-0.132829	0.000026
N	-0.095625	-1.229611	-0.000024
Sub-2			
C	-2.349255	-0.070467	0.000127
C	-1.758197	-1.309279	0.000199

C	0.31946	-0.371689	-0.000172
C	-0.121857	0.928221	-0.00014
C	-1.563089	1.097315	-0.000292
H	-2.354626	-2.216017	0.000587
H	-1.99458	2.089556	-0.000306
Cl	2.117744	-0.669206	0.000043
H	-3.434225	-0.003781	0.000396
N	-0.381536	-1.499923	-0.000226
C	0.738777	2.149786	0.00018
H	1.799263	1.907438	-0.000215
H	0.528874	2.778074	-0.878599
H	0.529351	2.777371	0.879574
Sub-5			
C	1.775981	-0.006451	0.000036
C	1.033663	-1.162267	0.000016
C	-0.923889	-0.014884	0.000136
C	-0.324252	1.21455	0.000044
C	1.115604	1.247935	0.000341
H	1.533351	-2.127377	-0.000254
H	1.662575	2.181431	0.000073
Cl	-2.736761	-0.040383	-0.000168
N	-0.357764	-1.216165	0.000365
H	-0.919512	2.116802	-0.000295
C	3.277398	-0.069021	-0.000273
H	3.632808	-1.102267	-0.000335
H	3.696359	0.43596	-0.880001
H	3.696672	0.435946	0.879319
Sub-6			
C	0.76226	0.00243	0.000001
C	-0.006558	-1.159858	0.000000
C	-1.960698	0.004529	0.000000
C	-1.325014	1.228528	0.000000
C	0.064806	1.28085	0.000000
H	0.500189	-2.121129	0.000000
H	0.59998	2.218393	-0.000001
Cl	-3.735659	-0.055301	0.000000
N	-1.332653	-1.215881	0.000000
H	-1.914357	2.137278	-0.000001
C	2.213649	-0.03827	0.000000
F	2.803222	0.606499	1.080082
F	2.744476	-1.289941	0.000000
F	2.803221	0.606498	-1.080083
Sub-7			

C	1.342246	-0.199004	-0.254274
C	0.534293	-1.290084	-0.067158
C	-1.336435	-0.012008	-0.022802
C	-0.662281	1.16604	-0.181499
C	0.769829	1.11011	-0.295538
H	0.978387	-2.278483	0.021839
H	1.33671	1.958543	-0.662229
Cl	-3.130386	0.083931	0.150041
N	-0.842778	-1.251089	0.037591
H	-1.200438	2.103364	-0.210745
C	2.826606	-0.341355	-0.340372
H	3.120125	-1.389755	-0.233279
H	3.195094	0.021567	-1.312376
O	3.503206	0.414939	0.688182
H	2.814923	0.993843	1.047359
Sub-8			
C	1.749622	0.948934	-0.000008
C	1.464229	-0.394272	-0.000007
C	-0.775193	0.054537	-0.000003
C	-0.624333	1.413048	-0.000002
C	0.721842	1.921229	0.00005
H	0.928994	2.98209	-0.000165
Cl	-2.476042	-0.576086	-0.000003
N	0.156751	-0.894268	0.000006
H	-1.487446	2.063522	0.000003
H	2.791481	1.25886	-0.000021
C	2.525798	-1.449015	-0.000001
H	2.431862	-2.104034	-0.877205
H	3.526866	-1.009892	-0.000034
H	2.431904	-2.103986	0.877246

References

1. Frisch MJ, Trucks GW, Schlegel HB *et al.* Gaussian 16 rev. A.03. Wallingford, CT; 2016.
2. Stephens PJ, Devlin FJ, Chabalowski CF *et al.* Ab initio calculation of vibrational absorption and circular dichroism spectra using density functional force fields. *J Phys Chem* 1994; **98**: 11623-11627.
3. Hermosilla L, Calle P, García de la Vega JM *et al.* Density functional theory predictions of isotropic hyperfine coupling constants. *J Phys Chem A* 2005; **109**: 1114-1124.
4. Humphrey W, Dalke A, Schulten K. Vmd: Visual molecular dynamics. *Journal of Molecular Graphics* 1996; **14**: 33-38.
5. Small-angle scattering of x-rays by a. Guinier and g. Fournet. *Acta Crystallographica* 1956; **9**: 326-326.
6. Kotlarchyk M, Chen SH. Analysis of small angle neutron scattering spectra from polydisperse interacting colloids. *J Chem Phys* 1983; **79**: 2461-2469.
7. Malmberg CG, Maryott AA. Dielectric constant of water from 0 °c to 100 °c. *J Res Nat Bureau Standards* 1956; **56**: 2641.
8. Hayter JB, Penfold J. An analytic structure factor for macroion solutions. *Mol Phys* 1981; **42**: 109-118.
9. Hansen J-P, Hayter JB. A rescaled msa structure factor for dilute charged colloidal dispersions. *Mol Phys* 1982; **46**: 651-656.
10. Berr SS. Solvent isotope effects on alkytrimethylammonium bromide micelles as a function of alkyl chain length. *J Phys Chem* 1987; **91**: 4760-4765.
11. Percus JK, Yevick GJ. Analysis of classical statistical mechanics by means of collective coordinates. *Physical Review* 1958; **110**: 1-13.
12. Mani T. Molecular qubits based on photogenerated spin-correlated radical pairs for quantum sensing. *Chem Phys Rev* 2022; **3**: 021301.
13. Hayashi H, Sakaguchi Y, Wakasa M. Magnetic field effects and spin dynamics of radical reactions in solution. *Bull Chem Soc Jpn* 2001; **74**: 773-783.
14. Liu G, Khlobystov AN, Charalambidis G *et al.* N@c60–porphyrin: A dyad of two radical centers. *J Am Chem Soc* 2012; **134**: 1938-1941.
15. Schleef D, Parry M, Tu S-J *et al.* Application of geometric probability techniques to the evaluation of interaction energies arising from a general radial potential. *J Math Phys* 1999; **40**: 1103-1112.
16. Feskov SV, Rogozina MV, Ivanov AI *et al.* Magnetic field effect on ion pair dynamics upon bimolecular photoinduced electron transfer in solution. *J Chem Phys* 2019; **150**: 024501.
17. Kira A, Thomas JK. Equilibriums between triplet states of aromatic hydrocarbons. *J Phys Chem* 1974; **78**: 196-199.
18. Das PK, Encinas MV, Scaiano JC. Laser flash photolysis study of the reactions of carbonyl triplets with phenols and photochemistry of p-hydroxypropiophenone. *J Am Chem Soc* 1981; **103**: 4154-4162.
19. Piñero L, Calderón X, Rodríguez J *et al.* Spectroscopic and electrochemical properties of 2-aminophenothiazine. *J Photochem Photobiol A Chem* 2008; **198**: 85-91.
20. Saucin M, Van de Vorst A. Photodynamic potentialities of some phenothiazine derivatives. *Radiation and Environmental Biophysics* 1980; **17**: 159-168.
21. Alkaitis SA, Beck G, Graetzel M. Laser photoionization of phenothiazine in alcoholic and aqueous micellar solution. Electron transfer from triplet states to metal ion acceptors. *J Am Chem Soc* 1975; **97**: 5723-5729.
22. Hester RE, Williams KPJ. Free radical studies by resonance raman spectroscopy: Phenothiazine,

10-methylphenothiazine, and phenoxazine radical cations. *J Chem Soc, Perkin Trans 2* 1981: 852-859.
23. Barra M, Calabrese GS, Allen MT *et al.* Photophysical and photochemical studies of phenothiazine and some derivatives: Exploratory studies of novel photosensitizers for photoresist technology. *Chem Mater* 1991; **3**: 610-616.
24. Steiner UE, Ulrich T. Magnetic field effects in chemical kinetics and related phenomena. *Chem Rev* 1989; **89**: 51-147.
25. Weller A, Nolting F, Staerk H. A quantitative interpretation of the magnetic field effect on hyperfine-coupling-induced triplet fromation from radical ion pairs. *Chem Phys Lett* 1983; **96**: 24-27.
26. Okada T, Yago T, Takamasu T *et al.* Cage lifetimes of ionic liquids as studied by the magnetic field effect probe. *Phys Chem Chem Phys* 2012; **14**: 3490-3497.
27. Megerle U, Lechner R, König B *et al.* Laboratory apparatus for the accurate, facile and rapid determination of visible light photoreaction quantum yields. *Photochem Photobiol Sci* 2010; **9**: 1400-1406.
